# Interrogation on the Cellular Nano-Interface and Biosafety of Repeated Nano-Electroporation by Nanostraw System

**DOI:** 10.3390/bios12070522

**Published:** 2022-07-13

**Authors:** Aihua Zhang, Jiaru Fang, Ji Wang, Xi Xie, Hui-Jiuan Chen, Gen He

**Affiliations:** 1 State Key Laboratory of Optoelectronic Materials and Technologies, Guangdong Province Key Laboratory of Display Material and Technology, School of Electronics and Information, Technology, Sun Yat-sen University, Guangzhou 510006, China; zhangaih3@mail2.sysu.edu.cn (A.Z.); fangjr9@mail2.sysu.edu.cn (J.F.); xiexi27@mail.sysu.edu.cn (X.X.); 2 The First Affiliated Hospital, Sun Yat-sen University, Guangzhou 510080, China; wangj683@mail.sysu.edu.cn; 3 Guangzhou Municipal and Guangdong Provincial Key Laboratory of Molecular Target & Clinical Pharmacology, The NMPA and State Key Laboratory of Respiratory Disease, School of Pharmaceutical Sciences and the Fifth Affiliated Hospital, Guangzhou Medical University, Guangzhou 511436, China

**Keywords:** nanostraw device, nano-electroporation, repeated electroporation, cellular safety, microfluidic device

## Abstract

Cell perforation is a critical step for intracellular drug delivery and real-time biosensing of intracellular signals. In recent years, the nanostraws system has been developed to achieve intracellular drug delivery with minimal invasiveness to the cells. Repeated cell perforation via nano-system could allow delivery of multiple drugs into cells for cell editing, but the biosafety is rarely explored. In this work, a nanostraw-mediated nano-electroporation system was developed, which allowed repeated perforation of the same set of cells in a minimally invasive manner, while the biosafety aspect of this system was investigated. Highly controllable fabrication of Al_2_O_3_ nanostraw arrays based on a porous polyethylene terephthalate (PET) membrane was integrated with a microfluidic device to construct the nanostraw-electroporation system. The pulse conditions and intervals of nano-electroporation were systematically optimized to achieve efficient cells perforation and maintain the viability of the cells. The cells proliferation, the early apoptosis activities after nanostraw-electroporation and the changes of gene functions and gene pathways of cells after repeated nano-electroporation were comprehensively analyzed. These results revealed that the repeated nanostraw-electroporation did not induce obvious negative effects on the cells. This work demonstrates the feasibility of repeated nano-electroporation on cells and provides a promising strategy for future biomedical applications.

## 1. Introduction

The cell is the basic component unit of the organism, and the normal operation of organelles and biological components in the cell ensures the normal operation of life activities. Studies on cellular function and physiological behavior provide an important theoretical basis for the diagnosis and treatment of diseases at the cellular level [1,2]. In the field of biomedicine, the development of intracellular detection techniques, including the detection of intracellular biochemical molecules and the electrical signal inside and outside the cell membrane, provide technical support for the investigation of cell functions and physiological activities [3,4]. On the other hand, intracellular drug delivery technology also plays a key role in influencing and manipulating cell function and fate. Accurate and repeatable drug delivery at different subcellular locations and organelles within cells offers potential therapeutic approaches for cell-level precision therapy, and even for genetic modification therapy of human diseases [5,6]. The key for both intracellular detection and intracellular drug delivery rely on the strategy of passing across the natural barrier of the cell membrane with minimal disruption to the cell [7].

At present, the methods for extracellular substances crossing the cell membrane are generally classified as vectors-mediated and membrane disruption-mediated [8]. The vectors-mediated approach is mainly based on the package of external molecules into the vectors, followed by molecules-carried vectors entering into the cell through endocytosis or membrane fusion process [9,10]. Vectors mainly include viral vectors and non-viral vectors. The main issue of the vectors-mediated method is that it is often dependent on specific cell types and packed molecules, and many efficient vectors such as viruses may cause cellular cytotoxicity and biosafety concerns [11]. The membrane destruction-mediated methods require the enhancement of the permeability of the cell membrane temporarily with the assistance of external forces [12,13]. This strategy perforates the membrane directly, and there is less restriction on the target cell types or to-be-delivered molecules. For delivery, the membrane destruction method could mediate a broad range of cargoes, such as DNA, siRNA, proteins, nanodrugs and nanoparticles, enter into the cell through the opening holes on the cell membrane [14,15]. On the other hand, the extraction and detection of intracellular substances also often benefit from the membrane destruction method since it allows the insertion of nanoprobes or nanoelectrodes into cells [16,17]. The membrane destruction-mediated methods mainly include microinjection and electroporation [18]. Microinjection techniques cannot satisfy high-throughput delivery into the cells [19]. Conventional bulk electroporation usually requires a high electric potential, creating extensive holes in the cell membrane, which can easily cause cell damage and even cell death [1].

Compared with conventional bulk electroporation, nano-electroporation technology can restrict the electric field into small nano-channels due to the localization effects of nanostructures [20,21]. Thus, the critical potential to cause cell membrane perforation can be significantly reduced and cell perturbation during electroporation can be minimized [2]. Among them, hollow nanostraw arrays have recently emerged as an effective tool for nano-electroporation, allowing delivery of biomolecules through their internal channels due to their unique hollow structures [22,23]. The nanostraw arrays have achieved reasonable success on high-throughput intracellular drug delivery [23]. A key advantage of nanostraws is that intracellular delivery can be repeatedly performed on the same set of cells due to the underneath chemical solution being flexibly applied through the hollow channels [24]. The repeated perforation of cells would allow repeated delivery of the drug into cells with precise temporal control. In our previous work, we have explored the possibility of continuous nano-electroporation via the nanostraw system [25], yet the possibility and safety of repeated poration by the nanostraw system remained unexplored. While most of the studies on the biosecurity of cells after nanostraw-electroporation have simply proved the viability of cells, few studies have been systematically conducted to evaluate the biosafety aspect of repeated applications of nanostraw-electroporation. The gene function and gene pathways in cells after repeated nanostraw-electroporation especially remain unknown.

**Figure 1:**
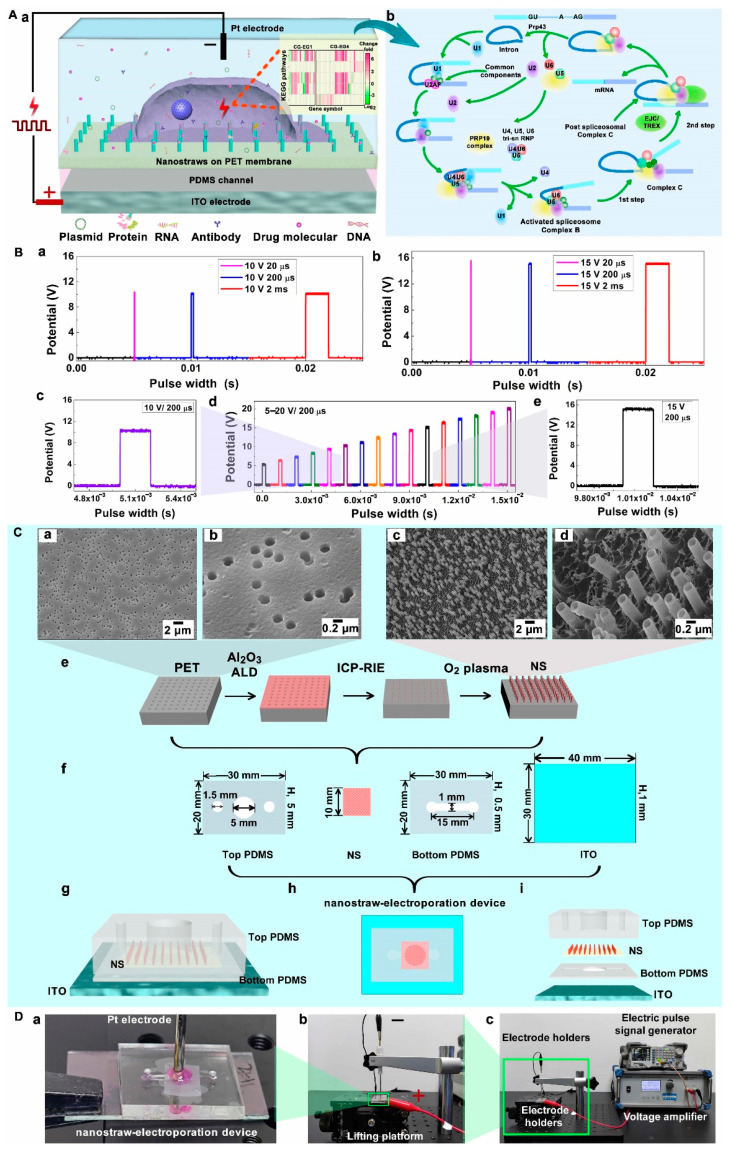
(**A**), Schematic illustration of the nanostraw-electroporation system. (**A-a**), Schematic diagram of nanostraw-electroporation on cells. Pt electrode and ITO (Indium Tin Oxide) electrode were set as the negative and positive electrode respectively. Insert, heat maps of the change folds of genes in their corresponding pathways. EG1, nanostraw-electroporation once, EG4, nanostraw-electroporation 4 times, CG, control group. (**A-b**), Schematic diagram of the spliceosome pathway. (**B**), The waveform electrical signals with different pulse widths (20 µs, 200 µs, and 2 ms. (**B-a**), 10 V, (**B-b**), 15 V) and potentials (**B-c**–**B-e**) generated by the electrical pulse signal generator and the voltage amplifier. (**B-d**), 5–20 V potential signals. (**B-c**,**B-e**), detail enlargement of 10 V (**B-c**) and 15 V (**B-e**) potential signal. (**C**) The SEM morphology of PET porous membrane (**C-a**,**C-b**) and the Al_2_O_3_ nanostraw-array on PET membrane (**C-c**,**C-d**). All the nanstraws were characterized with a 45-degree sample stage. (**C-e**), The schematic diagram of preparation process of Al_2_O_3_ nanostraw arrays. First, Al_2_O_3_ nanostraw array was fabricated based on PET porous membrane via ALD. Secondly, the top of PET membrane was etched by means of ICP-RIE technology. At last, the PET membrane was etched by oxygen plasma to obtain Al_2_O_3_ nanostraw array. (**C-f**,**C-i**), the assemble process (**C-f**,**C-h**) and 3 D schematic diagram (**C-g**,**C-i**) of nanostraw-electroporation device. (**C-f**,**C-h**), ITO glass, bottom PDMS layer, Al_2_O_3_ nanostraw-array and top PDMS pool layer were glued with uncured PDMS in bottom-up order. (**D**), Photographs of nanostraw-electroporation system and its partial enlargement. (**D-a**), the nanostraw-electroporation device, (**D-b**), the experimental setup of nanostraw-electroporation. (**D-c**), the construction of nanostraw-electroporation platform. Pt and ITO electrode were combined with the voltage amplifier and the electrical pulse signal generator via electrode holders.

In this work, we developed a nanostraw-electroporation system that allowed repeated perforation of the same set of cells in a minimally invasive manner, and the biosafety aspect of this system was investigated. Highly controllable fabrication of Al_2_O_3_ nanostraw arrays was conducted based on porous polyethylene terephthalate (PET) membrane as a template (Figure 1), and the nanostraws were assembled with a microfluidic device to construct the nanostraw-electroporation system. The pulse conditions and intervals of nanostraw-electroporation were systematically optimized using a fluorescent dye delivery assay so that repeated perforation of cells could be efficiently achieved, and the cell viability could be maintained. The cells proliferation, and early apoptosis activities after repeated nanostraw-electroporation at short intervals were investigated. What is more, the changes of gene functions and gene pathways of cells after repeated nanostraw-electroporation were comprehensively analyzed, where the results revealed that the repeated nanostraw-electroporation did not induce obvious negative effects on the cells. This work demonstrates the feasibility of repeated nanostraw-electroporation on cells and provides a promising strategy for future biomedical applications such as repeated delivery of gene materials or other biomolecules into the same set of cells for drug screening and gene editing.

## 2. Experimental Methods

### 2.1. Fabrication of Al_2_O_3_ Nanostraw-Array

The Al_2_O_3_ nanostraw was fabricated based on the track-etched polyethylene terephthalate (PET) porous membrane (Jiangbitou Manufacturing, Jinjiang, China), shown in Figure 1C-e. Firstly, the PET membrane was placed in the reaction chamber of the atomic layer deposition system (ALD, Cambridge Nanotech, Cambridge, MA, USA). The precursors of trimethylaluminum (TMA) and H_2_O (g) were injected into the reaction chamber in turn. The TMA would adsorb uniformly on the surface of the porous membrane and inside the pore as well. The reaction chamber was then purged with nitrogen to remove the excess TMA that was not adsorbed. When the water vapor was injected into the cavity, it would react with TMA. The Al_2_O_3_ atom layer was obtained via the reaction at 100 °C. The chamber was subsequently purged with nitrogen to remove the excess water vapor and methane, the gas produced via the reaction. After 300 cycles, a layer of Al_2_O_3_ with 50 nm thickness would be formed on the surface of the porous membrane and inside its pores.

Secondly, the Al_2_O_3_ layer on the top of the PET membrane was etched by inductively coupled plasma-reactive ion etching (ICP-RIE). The gas used for etching were, Cl_2_ 30 sccm (standard-state cubic centimeter per minute), SiCl_4_ 20 sccm, Ar 5 sccm. The etching power conditions were: ECR (electron cyclotron resonance) 300 W and RF (radio frequency) 60 W for 5 min. The nanostraw structure inside the PET was exposed. Thirdly, the PET membrane was etched with oxygen plasma (SPI Supplies, West Chester, PA, USA) to obtain a nanostraw-array with a specific height. The etching conditions were: 100 W for 30 min with 40 sccm of O_2_. The height of Al_2_O_3_ nanostraw was determined by the etching power and the etching time during the process of reactive oxygen plasma etching. The wall thickness of Al_2_O_3_ nanostraw was decided by the thickness of Al_2_O_3_ that was deposited via the ALD method, while the outer diameter of Al_2_O_3_ nanostraw depended on the pore size of the porous PET film.

### 2.2. Assemble of Nanostraw-Array Device

The device was constructed with the indium tin oxide (ITO) glass, the PDMS (polydimethylsiloxane) pools, and the Al_2_O_3_ nanostraw-array. The PDMS pools consisted of two parts: the top cell culture pond and the bottom drug molecules storage pool. They were prepared by specific molds with uncured PDMS (Syl Gard 184, Dow Corning, Midland County, MI, USA). The bottom layer was about 1 mm in height. There was a circular storage pool and a long channel in this layer. The width of the channel was 1 mm. The length was 15 mm. The diameter of the storage pool was 5 mm. The top layer was about 5 mm in height. It had a circular culture pool and two through-holes. Their diameters were 5 mm, 1.5 mm, 1.5 mm. respectively.

The uncured PDMS was used as the glue to stick the ITO glass, the bottom pool, the nanostraw-array, and the top pool together. When putting them in the oven at 80 °C for 4 h, the uncured PDMS would be cured, and the nanostraw-array device can be obtained (Figure 1C-f,C-i). Among them, the bottom PDMS channel layer was used to store drug molecules and gene plasmids to be delivered into cells. The top layer PDMS pool can be regarded as a cell culture pool, in which cells could undergo adhesion and proliferation. All devices were sterilized by 75% disinfectant alcohol (ethyl alcohol) and ultraviolet light, successively. Fibronectin (FN) was used to treat the nanostraw-array to enhance cells’ adhesion on the device.

### 2.3. Nanostraw-Electroporation

The electric equipment used in the nanostraw-electroporation system included an electrical pulse signal generator (RIGOL DG1032Z), a voltage amplifier (AGITEK ATA-214, Brædstrup, Denmark), and a digital storage oscilloscope (Tektronix TDS 2014C, Beaverton, UR, USA) (Figure S1 in Supplementary Materials). The electric pulse signal generator was combined with the voltage amplifier. The waveform electrical signals output by the electrical pulse signal generator were firstly amplified by the voltage amplifier. Then the signals would be detected and displayed via the digital storage oscilloscope (Figure 1B). The typical potential generated by the system was in the range of 10~20 V for cell perforation in our system. The commonly-used pulse width was 200 µs (Figure 1B-c–B-e).

Hela cells cultured in the device were refreshed with DMEM before electroporation. The bottom pool was rinsed with PBS. PI dye with 0.1 mg/mL was injected into the bottom pool through its channel. The top platinum column and bottom ITO glass were set as the negative electrode and the positive electrode, respectively (Figure 1D).

### 2.4. Characterizations

SEM (scanning electron microscope) was used to characterize the morphology of the Al_2_O_3_ nanostraw-array (Figure 1C-e), The Al_2_O_3_ nanostraw-array was sputtered with Au for 60 s before being observed under SEM. The height of the Al_2_O_3_ nanostraw was about 1 µm, the outer diameter was 450 nm and the sidewall thickness was 25 nm.

Calcein-AM, PI, 33342 were used to stain cells in the nanostraw-electroporation progress. The cell viability, PI delivery and cell nucleus were observed under fluorescence microscope (Leica, Sydney, Australia). Calcein-AM could be excited at 496 nm wavelength, PI could be excited at 535 nm wavelength, Hoechst 33342 could be excited at excitation 364 nm wavelength, respectively. Thus, the three filters above were selected to observe the fluorescence distribution of Hela cells.

### 2.5. COMSOL Simulation

**Figure 2:**
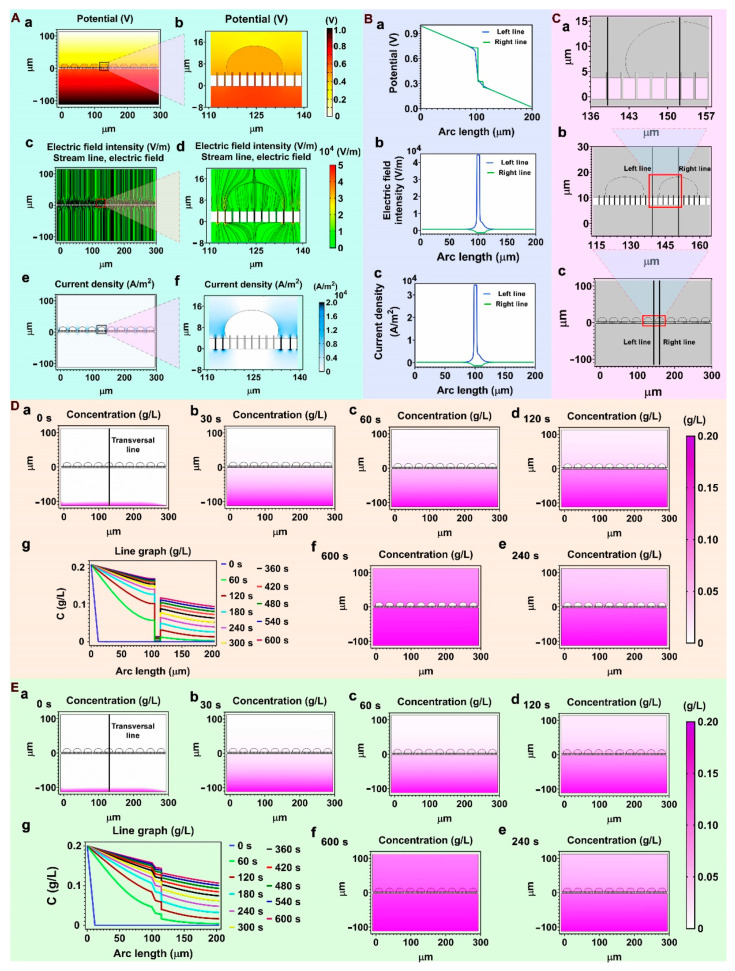
COMSOL simulation of nanostraw electroporation. (**A**), Profiles of the distribution of V, E, J and electric field stream line (**A-a**–**A-f**). The (**A-b**,**A-d**,**A-f**) were the local magnification of (**A-a**,**A-c**,**A-e**), respectively. (**B**), the value curves of V, E, J (**B-a**–**B-c**) on the vertical transversal lines in figure (**C**) (*X*-axis was the distance from the bottom electrode, *Y*-axis was the value of V, E and J). (**C**), the geometric graph of nanostraw-electroporation simulation model (**C-c**) and its partial enlarged graph (**C-a**,**C-b**), the left vertical line crossed the interior of nanostraw, the right vertical line crossed the interior of the nanostraw and the cell upside. (**D**), the drug molecules diffusion in nanostraw-array system within 600 s (**D-a**–**D-f**) before nanostraw-electroporation, a transversal line (**D-a**) crossed the nanosraw and the cell. (**D-g**), the diffusion concentration curve within 600 s on the transversal line (**a**) before cell perforation (time interval, 60 s). *X*-axis was the position on the transversal line, *Y*-axis was the concentration of drug molecules on the transversal line. (**E**), the drug molecules diffusion in nanostraw-array system within 600 s (**E-a**–**E-f**) after cell perforation, a transversal line (**E-a**) crossed the nanosraw and the cell. (**E-g**), drug molecules’ concentration changing along the transversal line (**E-a**) after nanostraw-electroporation with time in 600 s (time interval, 60 s). The distance from the bottom electrode and the drug molecules concentration on the transversal line were set as *X*-axis and *Y*-axis respectively.

In the Multiphysics simulation system of COMSOL, the physics of Electric Currents under the Electric Fields and Currents branch in the AC/DC module (steady state) was selected. For simplicity, we assumed the Al_2_O_3_ nanostraw was distributed in the PET film uniformly. According to the hole density of 7 × 10^5^ holes/cm^2^ given from the product manual of PET membrane, the center distance between the two Al_2_O_3_ nanostraw was about 3 μm after calculation. In the simulation system, Hela cells were simplified as ellipses with a long axis of 20 µm and a short axis of 15 µm (Figure 2C).

### 2.6. Statistical Analysis

All of the cells’ fluorescent images were analyzed with ImageJ. The cell viability was the proportion of living cells in all cells. The PI delivery efficiency was the proportion of delivered cells in all living cells. Statistics of the viabilities and efficiencies were plotted with Prism 8.0 (GraphPad). All histograms and the corresponding error bars were presented as mean ± SD (standard deviation).

The statistical progress in Table 1 and Table 2 was as follows: the number of cells stained by Hoechst 33342 (blue fluorescence) was counted as the total cell number (N), the number of cells stained by Calcein-AM (green fluorescence) was counted as the living cell number (N_green_), the number of dead cells was calculated as:

N_dead_ = N − N_green_. (1)

The number of cells stained by PI dye (red fluorescent cells) was counted as N_red_, and cell viability was calculated as:

Viability = N_green_/N. (2)

The cell transfection efficiency was calculated as:

Delivery = N_red_ − N_dead_/N. (3)

The digits in Table 1 and Table 2 were calculated as follows:

The mean and standard variance of the three sets of data in each group were calculated firstly. Then 4 significant digits were selected as the viability and Delivery efficiency value. Digits in Table 3 and Table 4 were calculated by the same way.

The statistical progress of the cell viability in Table 3 was referenced in Formula (2).

The statistical progress of the viability data in Table 4 was as follows:

Repeated nanostraw-electroporation within 8 min intervals could result in cell death, and the dead cells would be stripped from the device after incubating for 24 h. Therefore, we counted the number of living cells (stain by stained by Calcein-AM with green fluorescence) to assess the cells’ viability. The average number of cells in the control group device was counted as N_0_ and the number of cells after repeated nanostraw-electroporation treatment for 24 h under different time intervals was counted as N. The calculation progress of cell viability was as follows:

Viability = N/N_0_. (4)

### 2.7. Cell Proliferation Detection

The experimental procedures were as follows: certain numbers of Hela cells were planted in the 96-well plate. The numbers of cells were: 4 k, 8 k, 12 k, 20 k, 24 k and 28 k. When Hela cells were cultured for 12 h, CCK-8 was used to treat cells for 30 min to produce formazan in the culture medium. Then, the upper layer medium was absorbed and placed in a new 96-well plate. Next, the absorbance of these upper solutions at 450 nm was measured using a multi-detection microplate reader. Finally, the linear relationship between the numbers of Hela cells and the absorbances could be obtained. The number of cells was taken as the abaxial axis and the absorbance value of the upper liquid as the vertical axis (Figure S9).

### 2.8. RNA Extraction and Sequencing

After nanostraw-electroporation, Hela cells were cultured for another 24 h before RNA extraction. Then, the nanostraw-electroporation device was washed with PBS. Trizol solution (Thermo Fisher, Waltham, MA, USA) was added for cell lysis. The cell lysis solution was transferred to an Eppendorf tube with RNase-free (Thermo Fisher). Next, the trichloromethane (Aladdin, Shanghai, China) was added into the tube for layering of the lysis. The liquid supernatant was transferred into another Eppendorf tube with isopropanol (Aladdin) for RNA sedimentation. Under centrifugation, RNA would be sedimented completely at the bottom of the Eppendorf tube. Subsequently, the upper solution was removed away. RNA sedimentation was washed with 75% ethanol (Aladdin). It was centrifuged again, and the upper solution was removed. When the water and ethanol in the Eppendorf tube had completely evaporated, diethyl pyro carbonate (DEPC) water was added. There, the RNA extraction was complete. At last, RNA solution was stored at −80 °C. RNA sequencing was performed by the BGI Institute. The RNA sequencing results were presented in the interactive data mining system named Dr.Tom at the BGI Institute.

## 3. Results and Discussion

### 3.1. Simulation of Cell Electroperforation

In order to elucidate the mechanism of membrane perforation by nanostraws, COMSOL software was used to study the potential (V), electric field intensity (E), and current density (J) distribution in the nanostraw-electroporation system (Figure 2A,B). In this model, cells were simplified as ellipses that wrapped around and fully spread out on the nanostraw-array. The top PDMS pool layer and the bottom PDMS pond layer were simplified to rectangles. The Pt electrode and ITO electrode were set as the ground and the electric potential connection, respectively. Cell perforation occurred within microseconds, and the potential difference across the cytomembrane (transmembrane potential) was a critical value for membrane perforation. In the electric current physical model, the Laplace equation was used to calculate the distribution of E, V, and J at the electroporation moment (Formulas (5)–(7)) [25,26].

∇(σ·∇V) = 0(5)

E = −∇V(6)

J = σ E.(7)

“V” was the potential, and “σ” was the conductivity.

Parameters used in the simulation are shown in Table 5.

The transmembrane potential on the cell membrane could be calculated by Formula (8):

∆V_m_ = V_exl_ − V_inl.._ (8)

“V_exl_” was the outside surface of the cell membrane, “V_inl_” was the inside surface of the cell membrane. The simulated profiles of V, E, J distribution in Figure 2A show that there was an obvious V drop at the interface between cells and nanostraw-array (Figure 2A-a,A-b), the E and electric field streamlines were also localized at this position (Figure 2A-c,A-d), and the J was mainly distributed at the upper and lower openings of the nanostraw-array (Figure 2A-e,A-f). These V, E, J distributions showed that the membrane in contact with the nanostraw part was more likely to be electroporated. To further analyze the V, E, and J distribution at the interface between the nanostraw-array and the cell, two vertical transversal lines were plotted in the simulation model in Figure 2C. The left line crossed the interior of nanostraw, the right line crossed the interior of the nanostraw and the cell upside. *X*-axis was the distance from the bottom electrode on the lines, the *Y*-axis was the value of V, E, and J on the lines. The V, E, and J value curves along the vertical position were then plotted (Figure 2B). The curves of V values on the right line showed two obvious potential drops (Figure 2B-a).

The first one was at the interface of the nanostraw and the cytomembrane, accounting for 42% of the total potential. The other potential drop was at the top of the cell, accounting for 7% of the total potential. These potential drops were the transmembrane potential, which was the key for nanostraw-electroporation. As long as the potential drop (transmembrane potential) reached a critical value for membrane broken, nanostraw-electroporation would occur. In order to understand the transmembrane potential at the bottom and top of the membrane, seven vertical transversal lines that crossed the interior of the nanostraw and the cell were drawn in the simulation model (Figure S2A). We studied the transmembrane potential on the seven lines that have contact with the cell membrane, the results showing that there was a slight potential difference at the bottom of cell, but at the top of the cell, the transmembrane potential changed with the position of the transversal lines. The maximum potential was at the very top of the cell membrane, but it was still smaller than the potentials at the bottom of the cell (Figure S2B).

Therefore, in the nanostraw electroporation system, the part of the cells in contact with the nanostraw should be preferentially perforated. When the applied potential was too high, the top of the cell would also be perforated, resulting in excessive cell perforation and death. The relationship curves of electric field intensity (Figure 2B-b) and current density (Figure 2B-c) displayed similar trends. On the left curve, a peak value appeared at the bottom of the nanostraw and reached a stable value in the nanostraw inside the PET membrane. At the top of the nanostraw, the peak value dropped rapidly and then gradually decreased to a stable value. The E and J value curves showed that the value of E and J reached a peak both at the bottom and top of each nanostraw because of the convergence effect of nanostraws, which proved that the membrane on the nanostraw would be perforated preferentially. And the right lines show that, due to the presence of cells, J and E remained at a low value in the cell presence area. All these V, E, and J curves further proved that the nannostraw converging effect on V, E, and J could preferentially perforate the cytomembrane part that wrapped around the nanostraw. In addition, nanostraw-electroporation would also occur at the top of the cell with the applied potential increasing.

### 3.2. Simulation of Drug Molecules Diffusion

The diffusion process of drug molecules was also simulated by COMSOL software in the nanostraw-electroporation system. A 2D simulation model was selected to simulate and study drug molecules’ diffusion in the nanostraw electroporation system. The calculation formula of the concentration distribution was as follows [27]:

∂c∂t + ∇(−D∇c) = 0.(9)

“c” was the concentration of the drug molecules and D was the diffusion coefficient of the drug molecules. In the diffusion simulation process, the bottom channel was set as the drug molecules’ source, the concentration in it was supposed to be constant by default. Figure 2D-a–D-f showed the drug molecules’ diffusion process in the nanostraw system within 0–10 min. A detailed simulation of drug molecules’ concentration over time is shown in Figure S3 (time interval, 30 s). A vertical transversal line across the nanostraw and the cell upside was also plotted in the diffusion model. The distance from bottom electrode and the drug molecules’ concentration on the line was set as *X*-axis and *Y*-axis respectively. Drug molecules’ concentration changing within10 min along the transversal line was plotted in Figure 2D-g (time interval, 60 s). Due to the protection of the cytomembrane, there were significant differences in the diffusion of drug molecules into cells before and after electroporation. The drug molecules’ concentration inside the cell was lower than that outside before perforation. Drug molecules could be smoothly delivered into the cell after nanostraw-electroporation as shown in Figure 2E-a–E-g and Figure S4.

### 3.3. Optimization of Nanostraw-Electroporation Parameters

After the electrical and diffusion simulation, the appropriate parameters for nanostraw-electroporation on cells were examined. Hela cells were employed as model cells since this cell type has been widely employed in the field of the nanoneedle-cell interface. Propidium iodide (PI, Mw 668 Da, red fluorescence) dye was used as the to-be-delivered molecules since the PI delivery assay has been commonly applied as a standard assay to study the permeation of the cell membrane because PI dye normally could not enter into live cells, unless the cell membrane was perforated. The PI entering in the cellular cytosol could exhibit red fluorescence after binding with DNA in the cytosol. Therefore, the activity of cell membrane rupture could be identified by visualization of PI (red fluorescence) under a fluorescence microscope. On the other hand, PI could freely stain dead cells, since the membrane of dead cells was destructive.

In these experiments, 5 min after nanostraw-electroporation, cells were also stained by Calcein-AM (calcein-acetomethoxy methyl ester, green fluorescence dye), which has membrane permeability in living cells. When Calcein-AM entered into a cell, it would be cleaved to Cal xanthin by the intracellular esterase, combining with Ca^2+^ and producing a strong green fluorescence in the cell. Since dead cells lacked esterase, Calcein-AM could stain live cells only. Therefore, when Hela cells were perforated, red and green fluorescence would be simultaneously emitted under a fluorescence microscope.

The cell nuclei were stained with Hoechst 33342 to identify the total cell number. Hoechst 33342 (blue fluorescence) was a nuclear dye that could be visualized under fluorescence microscopy. The number rate of cells with green fluorescence indicated the living rate of cells, while the number rate of cells that displayed both green and red fluorescence represented the success rate of PI delivery into cells, which correlated to the cells that were electroporated by nanostraws with minimal invasiveness. The cells exhibited green fluorescence only without red fluorescence suggesting that the pulse conditions of the nanostraw-electroporation were too mild to perforate the cell membrane. On the other hand, if the cells exhibited red fluorescence only without green fluorescence, it meant that the pulse conditions of the nanostraw-electroporation were too invasive and cells were electrolyzed. The PI dye could be used as molecular cargo to quickly judge whether electroporation of the cell membrane was successful. In order to reduce the impact of PI toxicity, the excess PI after delivery was removed and was replaced with normal culture medium in 5 min after electroporation. The cells were immediately observed under a microscope to avoid cell death due to the toxicity of prolonged culture with PI dye. Therefore, the fluorescence of Hela cells after the nanostraws-mediated PI delivery assay could be used as a criterion for the screening of the nanostraw-electroporation conditions. The optimal conditions were based on the scenario in which the cells could maintain a high cell survival rate and high PI delivery rate at the same time after electroporation.

The electroporation parameters mainly included the value of the applied potential, the duration of the electrical perforation, and the width of the electric pulse. A large number of previous studies have proved that cells could live well on nanostraws and showed normal cell behavior [28]. Based on these studies, we focused on studying the effect of electrical stimulation or even repeated electrical stimulation on the behavior of cells cultured on nanostraws. In this work, electrical stimulation was the variable. Therefore, cells cultured on nanostraws without any electrical stimulation were set as the control group, and the electrical stimulation was set as a single factor to discover the effect of electrical stimulation more accurately.

In this work, the nanostraw-electroporation with a small pulse width (20 µs) and a large pulse width (2 ms) were studied (Figure 1B-a,B-b). The pulse frequency was set as 20 Hz according to our previous nanostraw-electroporation experience. The most optimal parameters, including electric potential, pulse width, and interval duration for nanostraw-electroporation, were explored in detail.

#### 3.3.1. Optimization of Potential and Pulse Duration

Firstly, the applied potential and the pulse duration to cause nanostraw-electroporation were investigated. The applied potential can be adjusted by the electrical pulse signal generator and voltage amplifier, where the output potential parameters at 5 V, 10 V, 15 V, and 20 V were examined, respectively. In our previous work, nanostraw-electroporation at a lower voltage of 10 V was favorable for cell perforation for a longer duration [25], while the application of higher voltages of 15 V or 20 V would be more suitable for short-term delivery due to higher voltages being better at ensuring cell perforation and facilitating delivery via electrophoresis. In addition, we employed electric pulses with 20-Hz frequency in this work rather than the 5 Hz we used in our previous work, because a higher frequency may also benefit delivery via electrophoresis during cell perforation. The duration parameters were controlled to 3 s, 10 s, 30 s, and 90 s.

**Figure 3:**
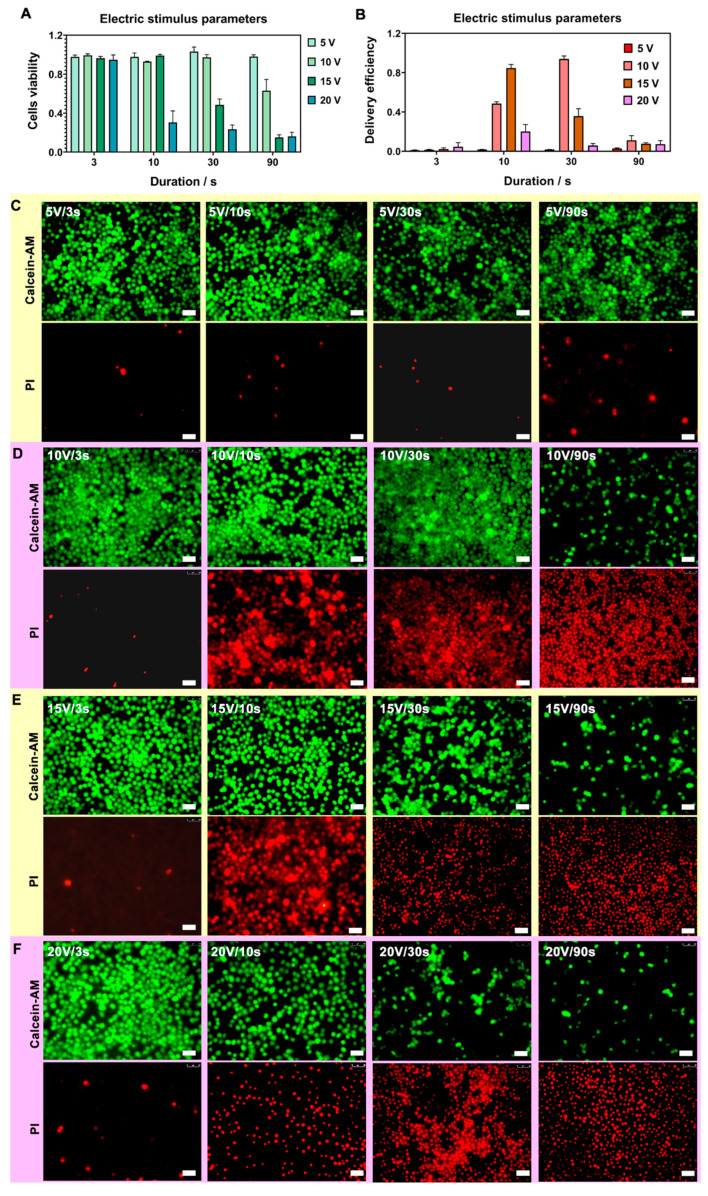
(**A,B**), Statistical analysis of nanostraw-electroporation vitality (**A**) and PI delivery efficiency (**B**) under four group of nanostraw-electroporation parameters (*n* = 3). The nanostraw-electroporation parameters were divided into four groups based on different potentials and electroporation durations. The first one was 5 V group with parameters of 5 V/3 s, 5 V/10 s, 5 V/30 s and 5 V/90 s. the second was 10 V group, included, 10 V/3 s, 10 V/10 s, 10 V/30 s and 10 V/90 s. The third 15 V group was 15 V/30 s, 15 V/30 s, 15 V/30 s and 15 V/90 s. The last 20 V group was, 20 V/3 s, 20 V/10 s, 20 V/30 s and 20 V/90 s. (**C**–**F**), The corresponding cell fluorescence diagrams after nanostraw-electroporation under these four groups of parameters ((**C**), 5 V group parameters. (**D**), 10 V group parameters. (**E**), 15 V group parameters. (**F**), 20 V group parameters). Live cells were stained by Calcein-AM (green fluorescence) after electroporation in all groups, and perforated cells were stained by the intracellular PI (red fluorescence), scale bar, 50 µm.

The results are shown in Figure 3A–D, where the fluorescence results indicate the delivery of PI dye (red) and cell viability after nanostraw-electroporation (Figures S5 and S6). The fluorescence results were also quantitatively analyzed, where the electric pulse of (10 V, 30 s) and (15 V, 10 s) at the condition of 200 μs pulse width and 20 Hz both showed successful intracellular delivery of PI dye and good cell viability. The cell viability data and the PI delivery efficiency data under each group of potential parameters are shown in Table 1 of the “Section 2.6”. We found that the factors (the electric potential, the pulse duration parameters) have a great influence on the efficiency of nanostraw-electroporation. For the lower electric potential, electroporation efficiency could be slightly improved by the increasing of pulse duration. For the higher electric potential, the manipulations of pulse duration failed to produce high efficiency and high viability perforation, and only contributed to either compromised or excessive electroporation.

#### 3.3.2. Optimization of Pulse Width

**Figure 4:**
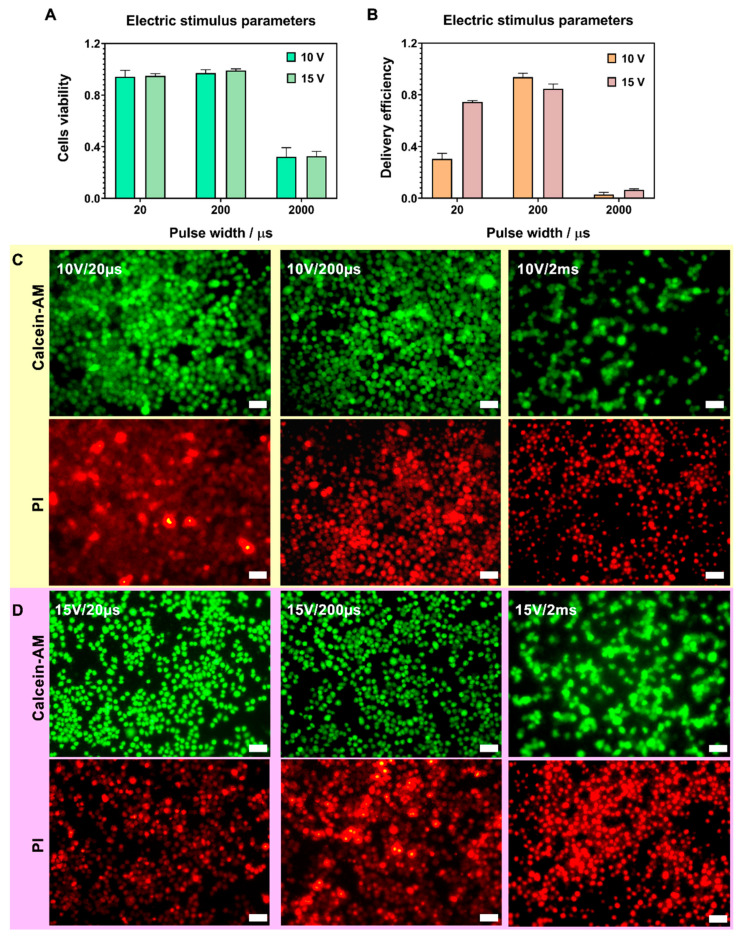
(**A**,**B**), Columnar analysis statistics of nanostraw-electroporation vitality (**A**) and PI delivery efficiency (**B**) under two new groups of nanostraw-electroporation parameters, *n* = 3. Parameters in group one were under 10 V potential with three kinds of pulse width—20 µs, 200 µs and 2 ms (2000 µs), the electroporation duration was 30 s. Parameters in group two were under 15 V potential with the same three kinds of pulse width—20 µs, 200 µs and 2 ms (2000 µs); the electroporation duration was 10 s. (**C**,**D**), The cells’ fluorescence diagrams after nanostraw-electroporation under the up two conditions. Live cells were stained by Calcein-AM (green fluorescence), and cells successfully electroporated were stained by the intracellular PI (red fluorescence), scale bar, 50 µm.

Next, the effects of applied pulse width at 20 μs, 200 μs, and 2 ms causing nanostraw-electroporation were investigated under the optimized conditions of (10 V, 30 s) and (15 V, 10 s). The statistical analysis diagrams of cells’ vitality and delivery efficiency are shown in Figure 4A,B. The corresponding fluorescence diagrams are shown in Figure 4C,D, where the fluorescence results indicate the delivery of PI dye (red) and cell viability after nanostraw-electroporation (Figure S7). The control group without electroporation was shown in Figure S8. The fluorescence results were also quantitatively analyzed, where nanostraw-electroporation conditions of (10 V, 30 s, 20 Hz) and (15 V, 10 s, 20 Hz) at the pulse width of 200 μs both showed successful intracellular delivery of PI and good cell viability. The cell viability data and the PI delivery efficiency data under each group of pulse width could be found in Table 2 of the “Section 2.6”.

The subsequent effects on cells after nanostraw-electroporation at optimal parameters were investigated, which included the influence on the subsequent proliferation and apoptosis of cells, as well as the influence on intracellular gene function and gene pathway.

### 3.4. Cell Proliferation and Apoptosis after Electroporation

The proliferation and apoptosis of Hela cells after nanostraw-electroporation were studied with the two groups of screened parameters of (10 V, 200 µs, 20 Hz, 30 s) and (15 V, 200 µs, 20 Hz, 10 s). Cell counting kit-8 (cck-8) was a tool for studying cell proliferation and viability. The WST-8 (2-(2-methoxy-4-nitrophenyl)-3-(4-nitrophenyl)-5-(2,4-disulfophenyl)-2H-tetrazolium-monosodium salt) in the kit could be reduced to highly water-soluble yellow formazan by dehydrogenase under the action of 1-methoxy PMS (1-methoxy-5-methylphenazinonium sulfate) in the cell. The amount of produced formazan was proportional to the number of living cells and could be measured by the absorbance at 450 nm with a microplate reader [29]. Therefore, cell proliferation could be analyzed by this characteristic.

Hela cells were plated in the nanostraw device and were cultured for 24 h. Then Hela cells were electroporated under the conditions of (10 V, 200 us, 20 Hz, 30 s) and (15 V, 200 us, 20 Hz, 10 s). After being cultured for another 24 h, cells were incubated with cck-8 solution for 2 h. The absorbance of upper mediate was measured, and the standard curve between the number of Hela cells and absorbance values is shown in Figure S9A. The absorbance values of the nanostraw-electroporation groups and the control group in Figure S9B further demonstrated that the cells could maintain high viability after treatments with nanostraw-electroporation.

Mitochondrial membrane potential was an important indicator of mitochondrial function and cell health [30]. In the process of cell apoptosis, the mitochondrial membrane potential gradient would decrease. JC-1 dye was a lipophilic cationic dye with green fluorescence. In healthy cells, JC-1 dye could enter and aggregate in negatively charged mitochondria, spontaneously forming red fluorescent aggregates. In unhealthy or apoptotic cells, due to the loss of mitochondrial membrane potential, JC-1 dye entered into the mitochondria less, and the formation of aggregates was insufficient, thus maintaining its original green fluorescence [31,32]. Therefore, the red/green fluorescence ratio in mitochondria was an assessment of the state of the mitochondria. The accumulation of JC-1 fluorescent dye in the mitochondria could be detected optically by fluorescence microscopy.

**Figure 5:**
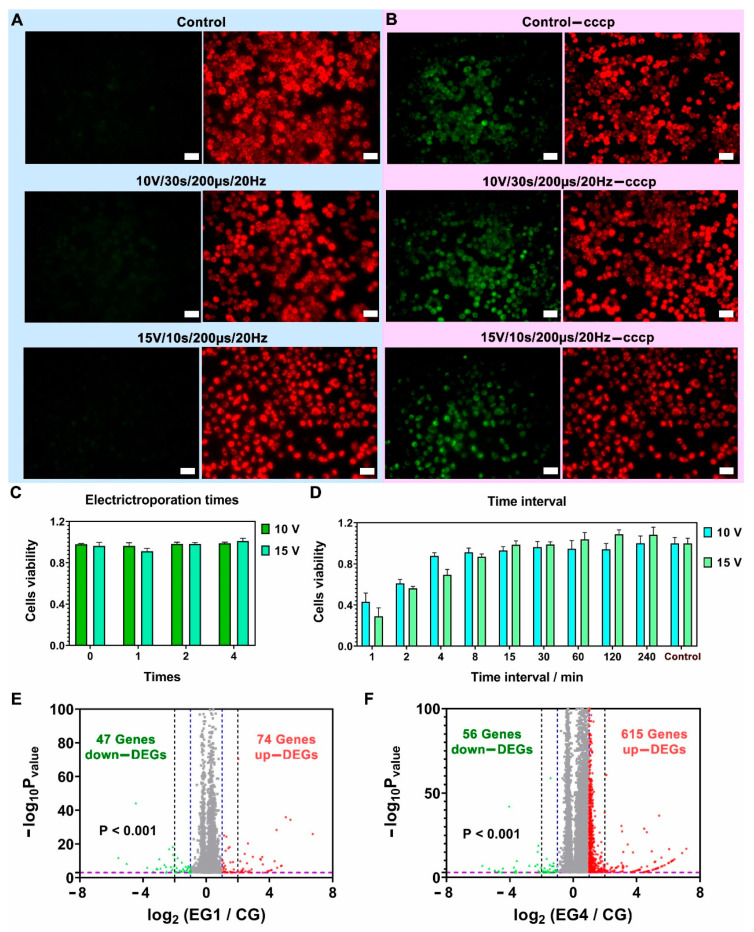
(**A**,**B**), cell apoptosis investigation after nanostraw-electroporation for 24 h, scale bar, 50 µm. Before electroporation, all cells were cultured in device for 24 h. (**A**), Cells stained with JC-1. The red fluorescence represented healthy mitochondria, showed healthy cells; Green fluorescence showed mitochondria in poor health, represented cells tend to wither early. (**B**), Hela cells were treated with CCCP (an inhibitor of mitochondrial electron transport chain), MMP would gradually decrease after CCCP treatment. The increased green fluorescence intensity in figure (**D**) showed the early apoptosis of cells. JC-1 dye was excited at 514 nm wavelength, JC-1 aggregate was excited at 585 nm wavelength. (**C**,**D**), Statistical analysis diagrams of cells’ vitalities after 0-, 1-, 2- and 4-cycles nanostraw-electroporation (**C**) and electroporation with different time intervals, 1 min ~ 240 min (**D**), *n* = 3. 10 V group (10 V, 200 µs, 20 Hz, 30 s), 15 V group (15 V, 200 µs, 20 Hz, 10 s). (**E**,**F**), Volcanic maps of genes with significant expression changes of EG1 ((**E**), nanostraw-electroporation once) and EG4 ((**F**), nanostraw-electroporation 4 times, the nanostraw-electroporation time interval was 4 h) compared with CG (control group), *n* = 3. Red scatters indicated genes with up-regulated expression, green scatters indicate genes with down-regulated expression, and gray scatters indicated genes with no significant difference in expression. The dashed blue line was the transversal of ±2 fold change, the dashed black line was the transversal of ±4 fold change, *p* < 0.001. Genes between the blue and black dash lines were changed between 2-fold and 4-fold, genes out of black dash lines were changed more than 4-fold. The dashed purple horizontal line was the transversal of *p* = 0.001.

In our detection of cell apoptosis experiments, Hela cells were first cultured for 24 h in the nanostraw-array device and were then electroporated with the conditions of (10 V, 200 µs, 20 Hz, 30 s) and (15 V, 200 µs, 20 Hz, 10 s). After being cultured for another 24 h, JC-1 was used for detecting cell apoptosis (Figure 5A). As a positive control, carbonyl cyanide m-chlorophenyl hydrazone (CCCP) was used, which was a chemical inhibitor of oxidative phosphorylation and affected the protein synthesis of mitochondria, leading to the gradual destruction of cells [33] (Figure 5B). The green fluorescence intensity of Hela cells was significantly enhanced compared with those without CCCP treatment. The early apoptosis studies on Hela cells showed that the nanostraw-electroporation did not significantly cause cell apoptosis.

### 3.5. Cell Repeated Electroporation

Repeated biomolecule delivery generally requires repeated cell perforation at safe electric conditions, thus, the viability and safety of cells after treatments of repeated nanostraw-electroporation were investigated. Firstly, the corresponding cell viabilities after treatments with a different number of cycles (0, 1, 2, and 4) of repeated nanostraw-electroporation with a 4 h-interval between each cycle were examined. Calcein-AM, PI dye, and Hoechst 33342 were used to stain and identify cells’ viability and cell number (Figure 5C and Figure S10). The results of repeated nanostraw-electroporation showed that Hela cells could maintain good viability even after repeated electrical stimulation four times within 24 h, suggesting 4 hours’ time interval was sufficiently long for cell membrane recovery after each nanostraw-electroporation. The cell viability data after 0, 1, 2, and 4 cycles of repeated nanostraw-electroporation can be found in Table 3 of the “Section 2.6”.

In the following experiments, the time interval between each cycle of nanostraw-electroporation was further shortened to evaluate cell viability at the more frequent perforation. The time intervals were set as 1 min, 2 min, 4 min, 8 min, 15 min, 30 min, 60 min, 120 min, and 240 min, respectively. The results are shown in Figure 5D, Figures S11 and S12. Under the conditions of (10 V, 200 μs, 20 Hz, 30 s), indicating that the viability of Hela cells was less compromised (>87.74 ± 3.10% viability) and remained safe when the time interval was longer than 4 min, and the viability was partially compromised (60.98 ± 3.16% viability) when the time interval was 2 min. The resultant cell death became worse, especially when the time interval was 1 min (43.09 ± 8.54% viability). The nanostraw-electroporation with a higher voltage (15 V) and a higher frequency (20 Hz) was more invasive and required a certain interval to maintain cell viability, compared to the previous work that used a lower voltage (10 V) and a lower frequency (5 Hz) [25]. Under the conditions of (15 V, 200 μs, 20 Hz, 10 s), the viabilities of Hela cells (<69.40 ± 5.24% viability) were affected when the time interval was less than 8 min, and the viabilities (98.63 ± 3.67% viability) were less compromised when the time interval was longer than 8 min.

These results indicate that repeated nanostraw-electroporation requires a certain time interval (>8 min) to maintain cell viability especially when intensive pulse conditions were applied. The cell viability data after four cycles of repeated nanostraw-electroporation under different time intervals are shown in Table 4 of the “Section 2.6”.

### 3.6. mRNA Sequencing and Analysis

In terms of repeated drug delivery, generally, a certain time interval longer than several hours is favorable in actual applications. Therefore, we further explored the alteration of gene functions or gene pathways in cells after repeated nanostraw-electroporation for one and four repeating cycles with a 4 hour time interval. The cells were treated with nanostraw-electroporation at the pulse condition of (10 V, 200 μs, 20 Hz, 30 s). The volcanic maps of genes with significant expression changes from the nanostraw-electroporation group with one cycle (EG1) and nanostraw-electroporation group with four cycles (EG4) were compared with the control group (CG, without electroporation), as shown in Figure 5E,F.

A total of 121 genes in EG1 and 671 genes in EG4 were significantly changed (*p* < 0.001), including 74 up-differentially expressed genes (up-DEGs) and 47 down-differentially expressed genes (down DEGs) in EG1, 615 up-DEGs and 56 down DEGs in EG4, corresponding to the red scatters and green scatters in Figure 5E,F. The blue and black dash lines were 2-fold change lines (significant change lines) and 4-fold change lines respectively. Genes within the blue dash lines were genes without significant changes, while the genes between blue dash line and black dash line were genes within 2-fold and 4-fold expression changes, and genes out of dash blue lines were genes changed more than 4-fold. In EG1, 48.76% changed genes were changed more than 4-fold, including 38 up DEGs and 21 down DEGs, while in EG4, 17.88% changed genes were changed more than 4-fold, including 89 up-DEGs and 31 down DEGs (*p* < 0.001). The volcano maps showed that the number of significantly changed genes after repeated nanostraw-electroporation for one cycle and four cycles accounted for only 0.057% and 0.32% of the total genes (210,503 genes) in the cell.

**Figure 6:**
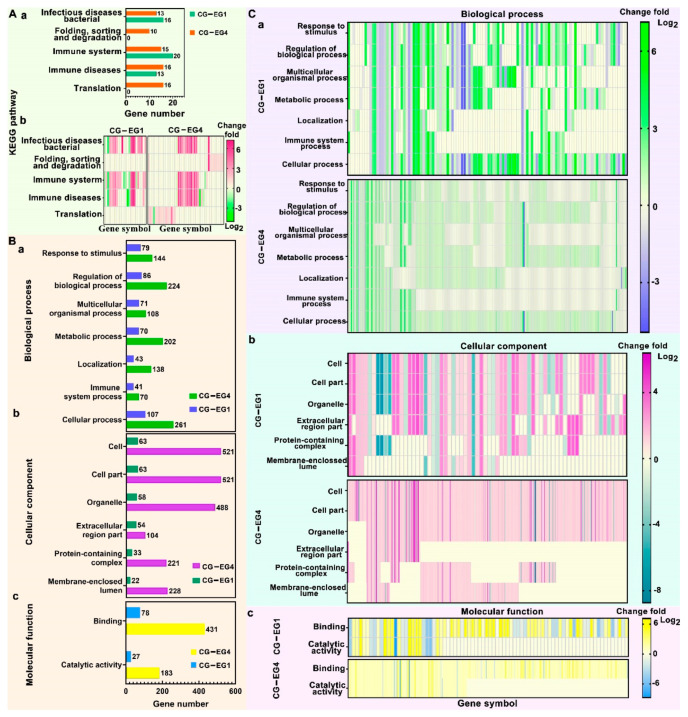
(**A**), KEGG pathway enrichment analysis in EG1 group and EG4 group compared with CG group, *n* = 3, *p* < 0.001. (**A-a**), the histogram of pathways changed and their corresponding genes’ number. (**A-b**), the heat maps of the change folds of genes in these corresponding pathways in EG1 and EG4 group compared with CG group. (**B**,**C**), GO term enrich analysis in EG1 group and EG4 group compared with CG group. GO term enrich analysis were divided into three parts, the biological process, the cellular component, and the molecular function, *n* = 3, *p* < 0.001. (**B**), the histogram of changed terms both in EG1 group and EG4 group compared with CG group, and their corresponding genes’ number. (**B-a**), the biological process, (**B-b**), the cellular component, (**B-c**), the molecular function. (**C**), heat maps of these changed genes’ change folds in their corresponding terms of EG1group and EG4 group compared with CG group. (**C-a**), the biological process, (**C-b**), the cellular component, (**C-c**), the molecular function.

The KEGG (Kyoto Encyclopedia of Genes and Genomes) pathway enrichment analysis and GO (Gene Ontology) enrichment analysis were also performed. In the KEGG pathway enrichment analysis, we counted all the pathways and the corresponding genes changed in both EG1 and EG4 (Figure 6A-a), and the changed pathways and genes’ changed folds were plotted in heat maps (Figure 6A-b). Similarly, all the changed GO terms and the corresponding number of genes were shown in Figure 6B, and the changed terms and the corresponding genes’ changed folds were plotted in heat maps (Figure 6C). The affected pathways in both EG1 and EG4 groups include “infectious diseases: bacteria”, “folding, sorting and degradation”, “immune system”, “immune diseases”. These results indicate that nanostraw electroporation may lead to the entry of external molecules into cells and induce cell diseases and immune responses. Other researchers have found that electric field stimulation can induce a cell fusion reaction, which may be related to the “folding, sorting and degradation” pathway in a cell.

The GO enrichment analysts included three terms—the biological process, the cellular component, and the molecular function. In the biological process term (Figure 6B-a), the top three processes that were significantly changed included the cellular process (changed genes increased from 107 genes in EG1 to 261 genes in EG4), the regulation of biological process (changed genes increased from 86 genes in EG1 to 224 genes in EG4), and the response to stimulus (changed genes increased from 79 genes in EG1 to 144 genes in EG4). The processes of the response to stimulus and the regulation of the biological process might be related to the cellular responding of external electric stimulation. In addition, the localization process might be associated with the perforated cell membrane part that underwent nanostraw-electroporation. In the cellular component term (Figure 6B-b), the top two cellular components were the cell and the cell part (changed genes increased from 63 genes in EG1 to 521 genes in EG4), the third component was the organelle (changed genes increased from 58 genes in EG1 to 488 genes in EG4), and the other components, such as the membrane-enclosed lumen, might be related to the self-repair of the cell membrane. In the molecular function term (Figure 6B-c), two functions were changed, where they were the binding (changed genes increased from 78 genes in EG1 to 431 genes in EG4) and the catalytic activity (changed genes increased from 27 genes in EG1 to 183 genes in EG4).

Melosh et al. found that bulk electroporation (500 V) resulted in larger cell disturbances compared to the nano-electro-injection (NEI) platform (30 V) [34]. Schmidler et al. also found that the electroporation of nanostraw did not interfere with gene expression in cells, whereas conventional electroporation altered the expression of more than 2000 genes [35]. The KEGG pathway and GO enrichment analysis showed that repeated nanostraw-electroporation did not cause obvious damage to the key cellular pathways, cellular processes, and functions. Although the number of changed genes increased with the increased cycles of nanostraw-electroporation, the change fold of these genes was mainly within 4-fold (82.12% of these genes).

## 4. Conclusions

In this work, an Al_2_O_3_ hollow nanostraw-array was prepared based on a nanoporous PET membrane as the template with a highly controllable fabrication procedure. A nanostraw-electroporation system with the nanostraw-array was constructed and used to screen the optimal parameters for repeated electroporation on Hela cells. The efficient delivery of PI proved the high efficiency of cell perforation by this system, and the biosafety aspects of cell vitality, cell proliferation, and apoptosis after nanostraw-electroporation were further verified. Gene sequencing analysis including the KEGG pathway enrichment analysis and the GO enrichment analysis were performed to comprehensively evaluate the gene changes due to nanostraw-electroporation, and the results suggested the repeated nanostraw-electroporation did not significantly alter the gene functions and gene pathways. The biosafety investigation on repeated nanostraw-electroporation provides a basis for the repeated delivery of intracellular drugs and offers experimental guidance on intracellular delivery for cell therapy.

## Figures and Tables

**Table 1:** The data of cell viability and PI delivery efficiency under each group of nanostraw-electroporation conditions.

Duration/s	Electrical Potential/V							
	5		10		15		20	
	Viability	Delivery	Viability	Delivery	Viability	Delivery	Viability	Delivery
3	97.76 ± 1.96%	1.12 ± 0.26%	99.41 ± 1.52%	1.46 ± 0.46%	96.36 ± 1.91%	2.15 ± 1.39%	94.84 ± 4.94%	4.61 ± 4.21%
10	97.73 ± 4.07%	1.77 ± 0.14%	92.92 ± 0.45%	48.62 ± 1.71%	96.36 ± 1.92	84.62 ± 3.74%	30.46 ± 11.83%	19.93 ± 7.33%
30	103.18 ± 4.69%	1.75 ± 0.04%	97.27 ± 2.89%	93.85 ± 3.14%	96.36 ± 1.93	35.77 ± 7.55%	23.40 ± 4.47%	5.84 ± 2.12%
90	98.03 ± 1.84%	2.85 ± 0.64%	63.04 ± 11.54%	11.04 ± 4.85%	96.36 ± 1.94	7.63 ± 1.11%	16.11 ± 4.26%	7.22 ± 3.64%

**Table 2:** The data of cell viability and PI delivery efficiency under each group of pulse width conditions.

Pulse Width/μs	Electrical Potential			
	10 V		15 V	
	Viability	Delivery	Viability	Delivery
20	94.21 ± 4.94%	30.45 ± 4.29%	94.85 ± 1.78%	74.41 ± 1.06%
200	97.04 ± 2.62%	93.73 ± 2.93%	99.07 ± 1.22%	84.62 ± 3.74%
2000	32.10 ± 7.14%	2.86 ± 1.68%	32.68 ± 3.72%	6.51 ± 0.75%

**Table 3:** The data of cell viability after 0, 1, 2, and 4 cycles of repeated nanostraw-electroporation.

Electroporation Cycles	Viability	
	10 V	15 V
0	97.87 ± 0.79%	96.18 ± 3.52%
1	96.28 ± 3.09%	91.18 ± 2.79%
2	98.23 ± 1.65%	98.12 ± 1.42%
4	98.91 ± 1.04%	101.02 ± 2.64%

**Table 4:** The data of cell viability after 4 cycles of repeated nanostraw-electroporation under different time intervals.

Time Interval/Min	Viability	
	10 V	15 V
1	43.09 ± 8.54%	29.01 ± 8.23%
2	60.98 ± 3.86%	56.22 ± 1.85%
4	87.74 ± 3.10%	69.40 ± 5.24%
8	91.25 ± 4.11%	86.96 ± 2.68%
15	93.03 ± 3.72%	98.63 ± 3.67%
30	96.26 ± 5.50%	98.92 ± 2.52%
60	94.59 ± 8.04%	103.77 ± 6.71%
120	94.20 ± 5.58%	108.74 ± 4.31%
240	100.11 ± 7.00%	108.28 ± 7.36%
Control	100.00 ± 5.70%	99.96 ± 4.95%

**Table 5:** Parameters of the 2 D Model.

Symbol	Value	Definition
d	6 nm	cell membrane thickness
σ_e_	0.2 S/m	electric conductivity of external medium
σ_i_	0.2 S/m	electric conductivity of cytoplasm
σ_m_	5 × 10^−7^ S/m	electric conductivity of cell membrane

## Supplementary Materials

The following supporting information can be downloaded at: https://www.mdpi.com/article/10.3390/bios12070522/s1, Figure S1: Photograph of the nanostraw-electroporation equipment, the electric pulse signal generator outputted the pre-defined waveform electrical signals, the voltage amplifier could amplify the signals, the digital storage oscilloscope could detect and display the signals. Figure S2: The geometric structure of nanostraw-electroporation simulation model (A) and the diagrams of COMSOL multi-physical simulation results on the 7 transvel lines (B). Figure S3: the diffusion simulation of drug molecules in nanostraw-array system from 0 s to 600 s without electroporation, time interval 30 s, scale bar 20 µm. Figure S4: the diffusion simulation of drug molecules in nanostraw-array system from 0 s to 600 s after electroporation, time interval 30 s, scale bar, 20 µm. Figure S5: cell fluorescence diagrams after nanostraw-electroporation under 5 V group parameters (5 V/3 s, 5 V/10 s, 5 V/30 s and 5 V/90 s) and 10 V group parameters (10 V/3 s, 10 V/10 s, 10 V/30 s and 10 V/90 s). Live cells were stained by Calcein-AM (green fluorescence), cells electroporated successfully were stained by the delivered PI dye (red fluorescence), cells’ nucleuses were stained with Hoechst 33342 (blue fluorescence), scale bar, 50 µm. Calcein-AM was excited at 496 nm wavelength, PI was excited at 535 nm wavelength, Hoechst 33342 was excited at excitation 364 nm wavelength. Figure S6: cell fluorescence diagrams after nanostraw-electroporation under 15 V group parameters (15 V/3 s, 15 V/10 s, 15 V/30 s and 15 V/90 s) and 20 V group parameters (20 V/3 s, 20 V/10 s, 20 V/30 s and 20 V/90 s). Live cells were stained by Calcein-AM (green fluorescence), cells electroporated successfully were stained by the delivered PI dye (red fluorescence), cells’ nucleuses were stained with Hoechst 33342 (blue fluorescence), scale bar, 50 µm. Calcein-AM was excited at 496 nm wavelength, PI was excited at 535 nm wavelength, Hoechst 33342 was excited at excitation 364 nm wavelength. Figure S7: cells fluorescence diagrams after nanostraw-electroporation under 10 V and 15 V potential with three kinds of pulse width, 20 µs, 200 µs and 2 ms (2000 µs). Live cells were stained by Calcein-AM (green fluorescence), cells electroporated successfully were stained by the delivered PI dye (red fluorescence), cells’ nucleus were stained with Hoechst 33342 (blue fluorescence). scale bar, 50 µm. Calcein-AM was excited at 496 nm wavelength, PI was excited at 535 nm wavelength, Hoechst 33342 was excited at excitation 364 nm wavelength. Figure S8: cells fluorescence diagrams without nanostraw-electroporation. Figure S9: A, the standard curve between the number of Hela cells and absorbance values. The numbers of cells were, 4 k, 8 k, 12 k, 20 k, 24 k, 28 k. the number of cells as the *X*-axis and the absorbance value of the upper liquid of cells as the *Y*-axis, n = 3. B, Cell proliferation diagram after cultured in device for 48 hours. At the 24th hour, cells were treated with nanostraw-electroporation under three types of nanostraw-electroporation parameters, 10 V, 30 s, 200 μs (red dots); 15 V, 10 s, 200 μs (purple dots); control group (blue dots), n = 4. Figure S10, cells fluorescence diagrams after nanostraw-electroporation after 0-, 1-, 2- and 4-times nanostraw-electroporation under 10 V, 200 µs, 20 Hz, 30 s and 15 V, 200 µs, 20 Hz, 10 s. Live cells were stained by Calcein-AM (green fluorescence), dead cells were stained by PI (red fluorescence), cells’ nucleus were stained with Hoechst 33342 (blue fluorescence). scale bar, 50 µm. Calcein-AM was excited at 496 nm wavelength, PI was excited at 535 nm wavelength, Hoechst 33342 was excited at excitation 364 nm wavelength. Figure S11, cells fluorescence diagrams after four times nanostraw-electroporation under 10 V, 200 µs, 20 Hz, 30 s with time intervals of, 1 min, 2 min, 4 min, 8 min, 15 min, 30 min, 60 min, 120 min, 240 min, and control group without nanostraw-electroporation. Live cells were stained by Calcein-AM (green fluorescence), dead cells were stained by PI (red fluorescence), cells’ nucleus were stained with Hoechst 33342 (blue fluorescence). scale bar, 50 µm. Calcein-AM was excited at 496 nm wavelength, PI was excited at 535 nm wavelength, Hoechst 33342 was excited at excitation 364 nm wavelength. Figure S12, cells fluorescence diagrams after four times nanostraw-electroporation under 15 V, 200 µs, 20 Hz, 10 s with time intervals of, 1 min, 2 min, 4 min, 8 min, 15 min, 30 min, 60 min, 120 min, 240 min, and control group without nanostraw-electroporation. Live cells were stained by Calcein-AM (green fluorescence), dead cells were stained by PI (red fluorescence), cells’ nucleus were stained with Hoechst 33342 (blue fluorescence). scale bar, 50 µm. Calcein-AM was excited at 496 nm wavelength, PI was excited at 535 nm wavelength, Hoechst 33342 was excited at excitation 364 nm wavelength.
