# Supplementary material for: Interrogation on the Cellular Nano-Interface and Biosafety of Repeated Nano-Electroporation by Nanostraw System
Source: Biosensors (Basel). 2022 Jul 13;12(7):522. doi: 10.3390/bios12070522 (PMC9313307; doi:10.3390/bios12070522)
Supplement: Supplementary file 1 [file biosensors-12-00522-s001.zip › biosensors-1796891-supplementary.pdf]

Supplementary Materials

Interrogation on the Cellular Nano-Interface and Biosafety of Repeated Nano-Electroporation by Nanostraw System

S1, The equipment used in this nanostraw-electroporation system.

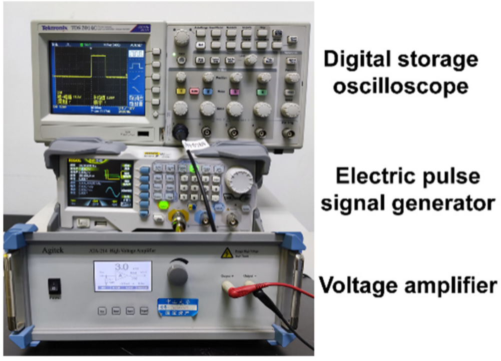

Figure S1. Photograph of the nanostraw-electroporation equipment, the electric pulse signal generator outputted the pre-defined waveform electrical signals, the voltage amplifier could amplify the signals, the digital storage oscilloscope could detect and display the signals.

S2, comsol simulation of the transmembrane potential on the transvel lines.

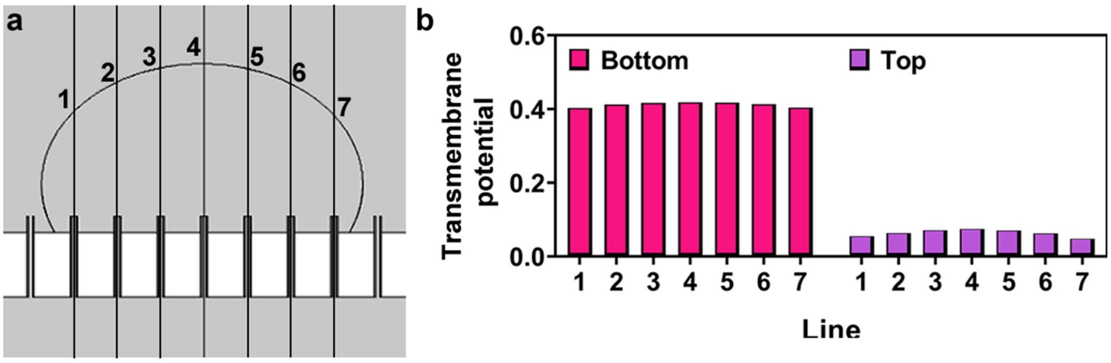

Figure S2. the geometric structure of nanostraw-electroporation simulation model (A) and the diagrams of COMSOL multi-physical simulation results on the 7 transvel lines (B).

S3. comsol simulation of drug molecules delivery whitin 0-10 min without nanostraw-electroporation.

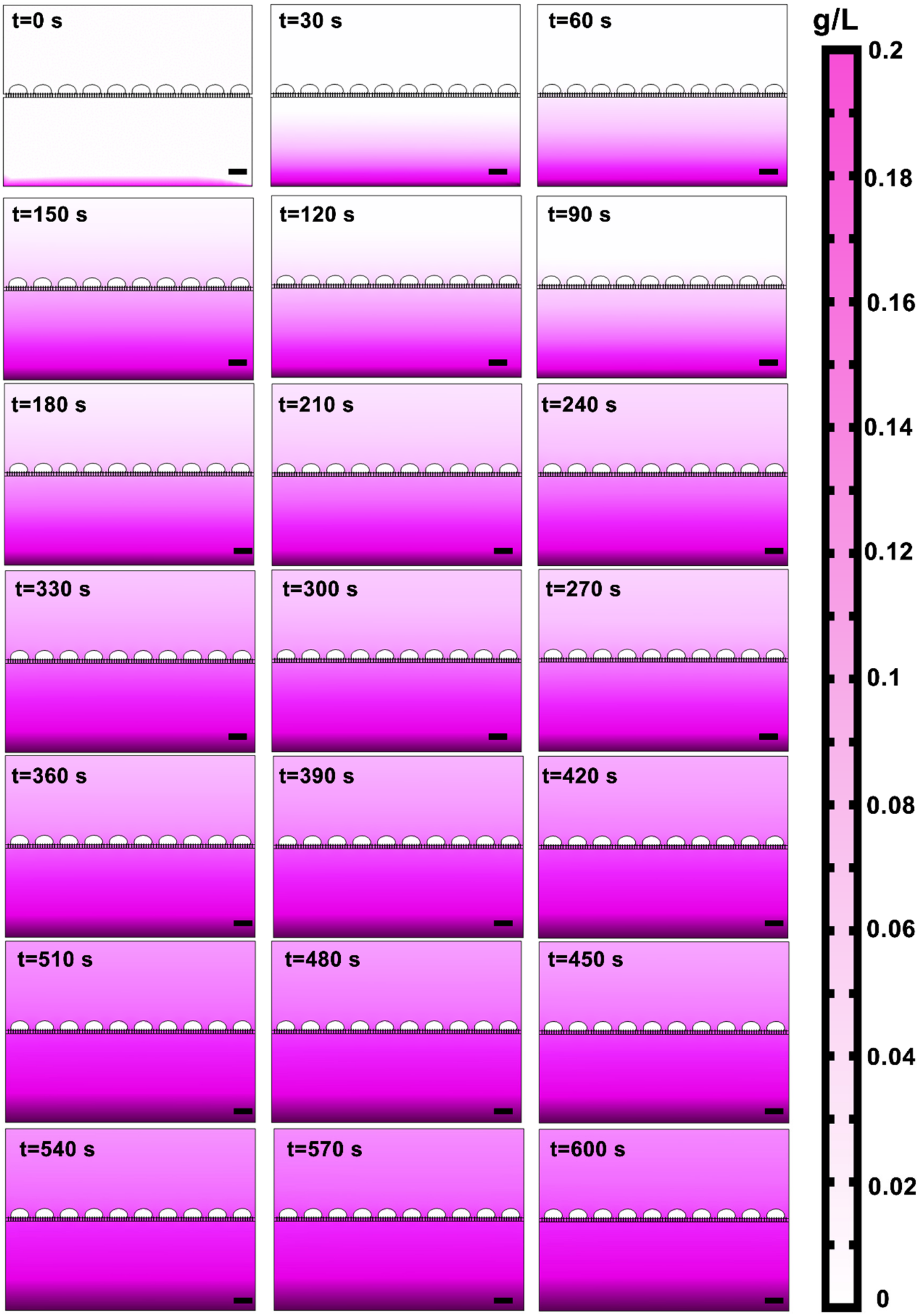

Figure S3. the diffusion simulation of drug molecules in nanostraw-array system from 0 s to 600 s without electroporation, time interval 30 s, scale bar 20 μm.

S4, comsol simulation of drug molecules delivery whitin 0-10 min after cell membrane electroporated.

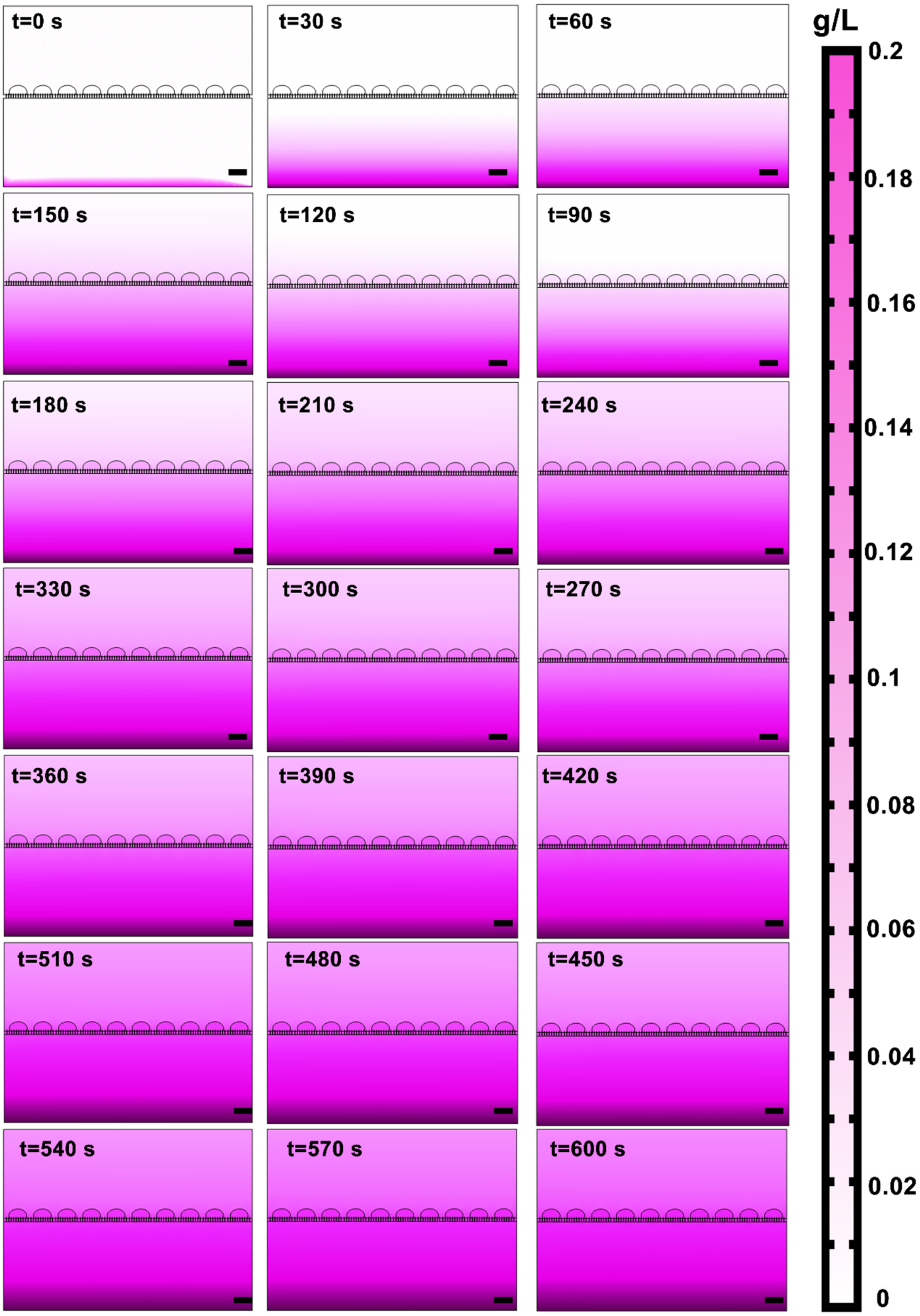

Figure S4. the diffusion simulation of drug molecules in nanostraw-array system from 0 s to 600 s after electroporation, time interval 30 s, scale bar, 20 μm.

S5, The detailed trichromatic fluorescence diagrams of Hela cells under the applied potential and the pulse duration, the potential was 5 V, 10 V, the pulse duration was 3 s, 10 s, 30 s, 90 s.

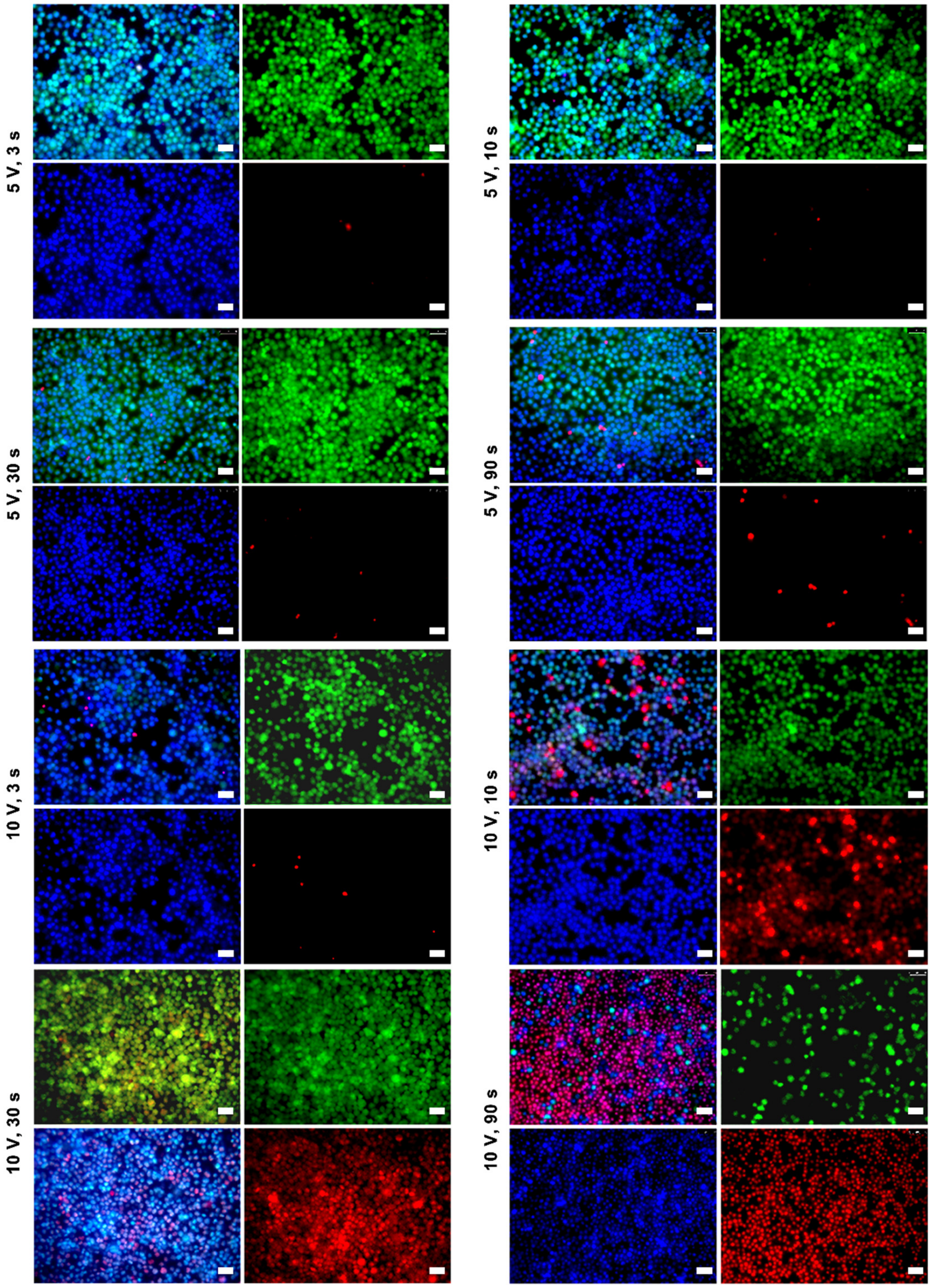

Figure S5. cell fluorescence diagrams after nanostraw-electroporation under 5 V group parameters (5 V / 3 s, 5 V / 10 s, 5 V / 30 s and 5 V / 90 s) and 10 V group parameters (10 V / 3 s, 10 V / 10 s, 10 V / 30 s and 10 V / 90 s). Live cells were stained by Calcein-AM (green fluorescence), cells electroporated successfully were stained by the delivered PI dye (red fluorescence), cells' nucleuses were stained with Hoechst 33342 (blue fluorescence), scale bar, 50 μm. Calcein-AM was excited at 496 nm wavelength, PI was excited at 535 nm wavelength, Hoechst 33342 was excited at excitation 364 nm wavelength.

S6, The detailed trichromatic fluorescence diagrams of Hela cells under the applied potential and the pulse duration, the potential was 15 V, 20 V, the pulse duration was 3 s, 10 s, 30 s, 90 s.

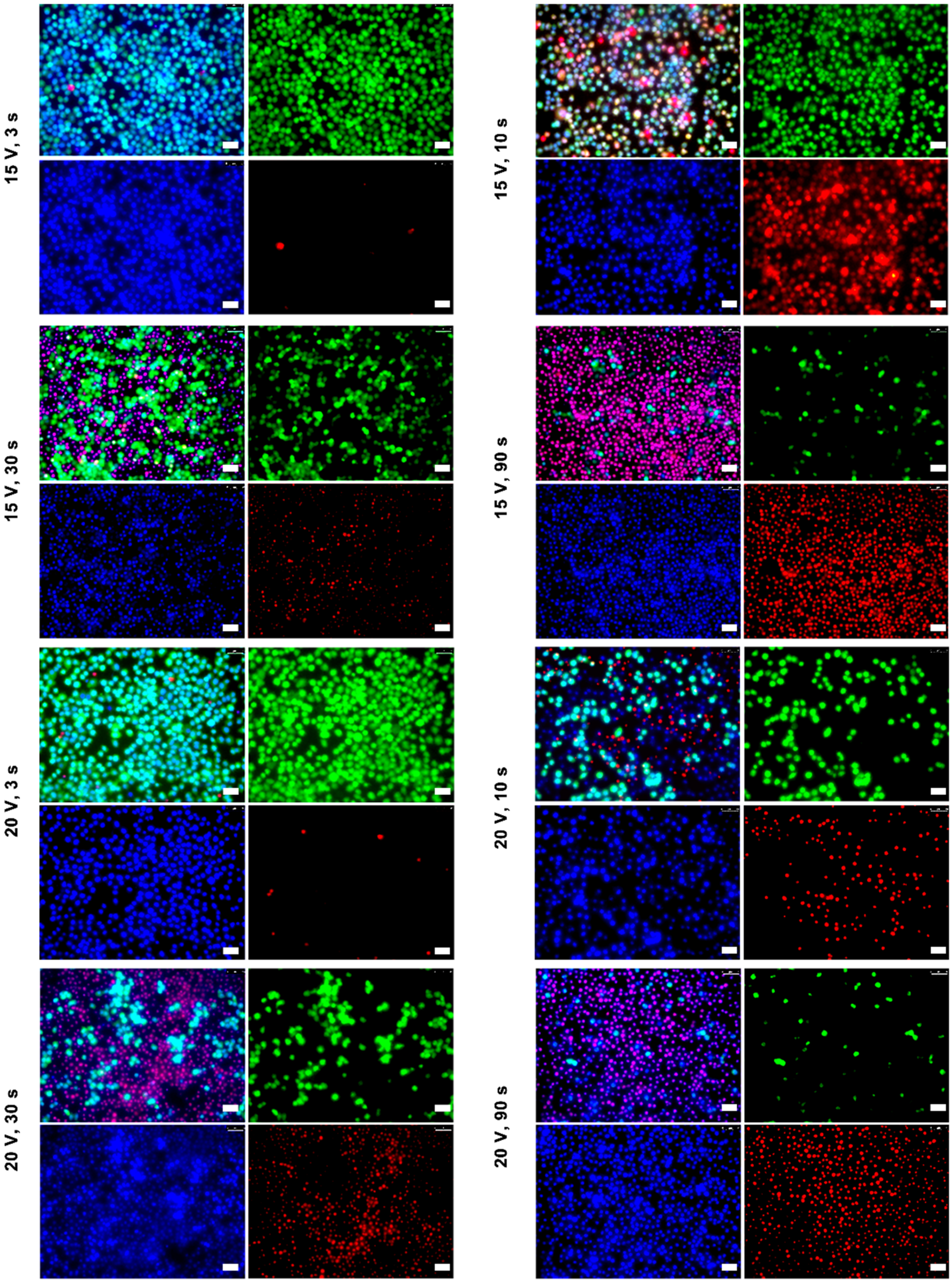

Figure S6. cell fluorescence diagrams after nanostraw-electroporation under 15 V group parameters (15 V / 3 s, 15 V / 10 s, 15 V / 30 s and 15 V / 90 s) and 20 V group parameters (20 V / 3 s, 20 V / 10 s, 20 V / 30 s and 20 V / 90 s). Live cells were stained by Calcein-AM (green fluorescence), cells electroporated successfully were stained by the delivered PI dye (red fluorescence), cells' nucleuses were stained with Hoechst 33342 (blue fluorescence), scale bar, 50 μm. Calcein-AM was excited at 496 nm wavelength, PI was excited at 535 nm wavelength, Hoechst 33342 was excited at excitation 364 nm wavelength.

S7, The detailed trichromatic fluorescence diagrams of Hela cells with the applied pulse width of 20 μs, 200 μs and 2 ms under the screened conditions of (10 V, 30 s) and (15 V, 10 s).

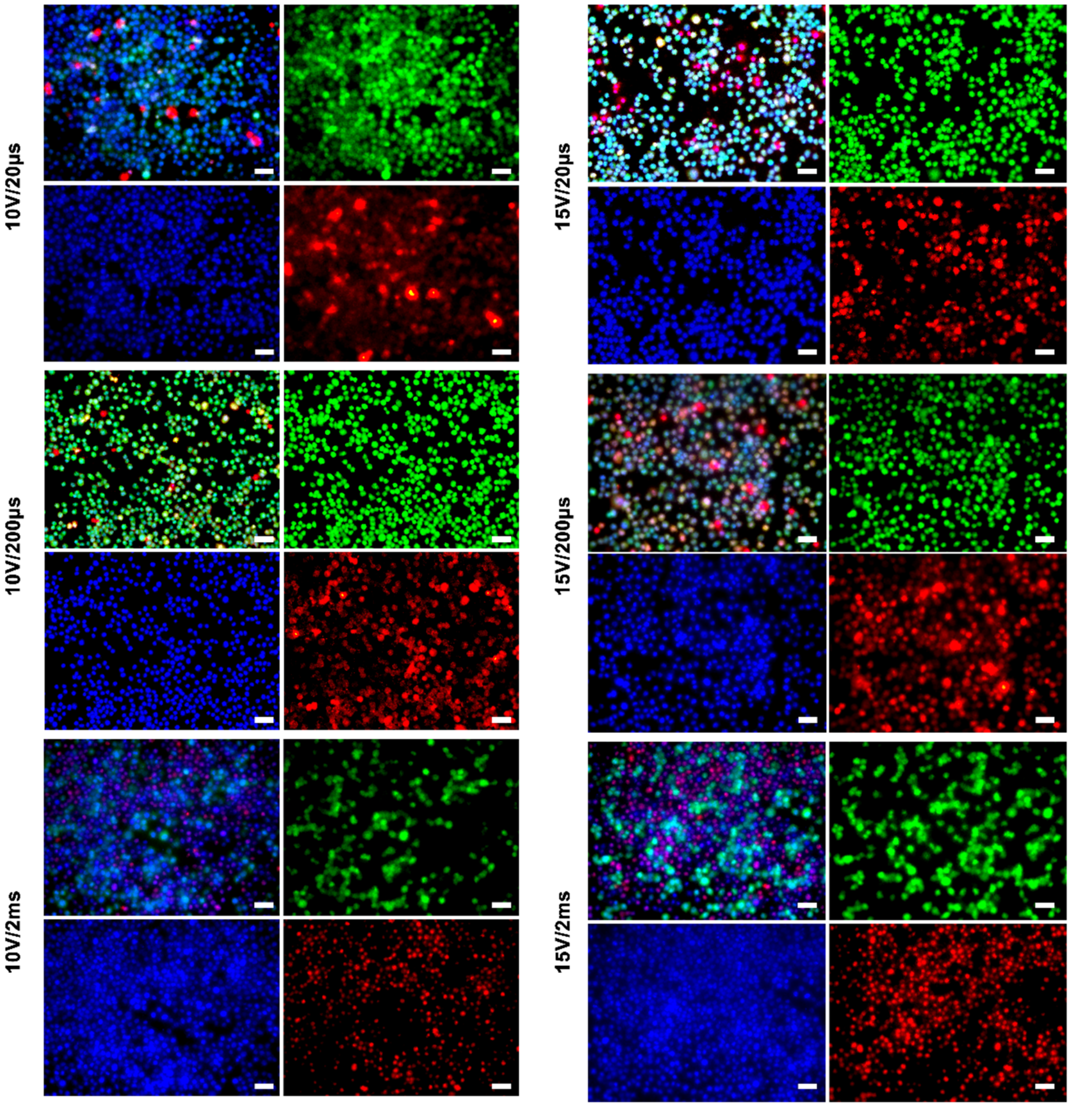

Figure S7. cells fluorescence diagrams after nanostraw-electroporation under 10 V and 15 V potential with three kinds of pulse width, 20 μs, 200 μs and 2 ms (2000 μs). Live cells were stained by Calcein-AM (green fluorescence), cells electroporated successfully were stained by the delivered PI dye (red fluorescence), cells' nucleus were stained with Hoechst 33342 (blue fluorescence). scale bar, 50 μm. Calcein-AM was excited at 496 nm wavelength, PI was excited at 535 nm wavelength, Hoechst 33342 was excited at excitation 364 nm wavelength.

S8, The detailed trichromatic fluorescence diagrams of Hela cells in control group without electroporation

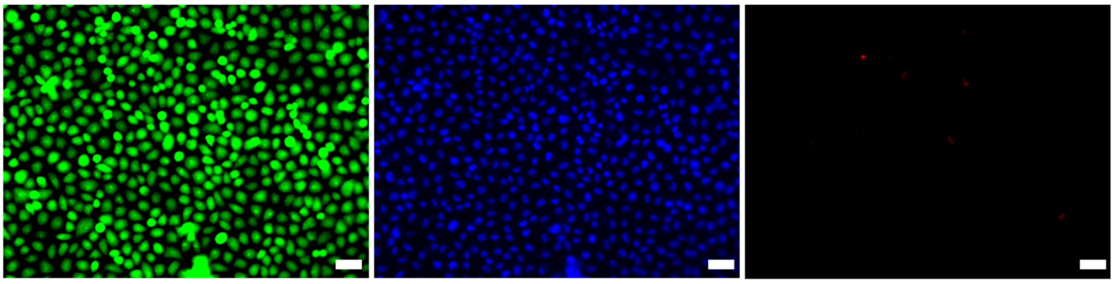

Figure S8. cells fluorescence diagrams without nanostraw-electroporation. Live cells were stained by Calcein-AM (green fluorescence), cells' nucleus were stained with Hoechst 33342 (blue fluorescence), dead cells were stained by PI dye (red fluorescence). scale bar, 50 µm. Calcein-AM was excited at 496 nm wavelength, PI was excited at 535 nm wavelength, Hoechst 33342 was excited at excitation 364 nm wavelength.

S9, the linear relationship between the number of Hela cells and absorbance values and the absorbance values of nanostraw-electroporation groups and control group.

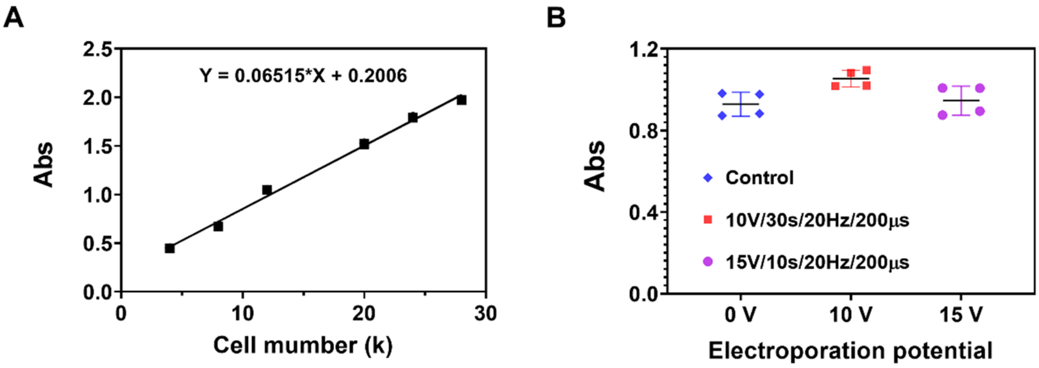

Figure S9. A, the standard curve between the number of Hela cells and absorbance values. The numbers of cells were, 4 k, 8 k, 12 k, 20 k, 24 k, 28 k. the number of cells as the X-axis and the absorbance value of the upper liquid of cells as the Y-axis, n=3. **B,** Cell proliferation diagram after cultured in device for 48 hours. At the 24th hour, cells were treated with nanostraw-electroporation under three types of nanostraw-electroporation parameters, 10 V, 30 s, 200 µs (red dots); 15 V, 10 s, 200 µs (purple dots); control group (blue dots), n=4.

S10, The detailed trichromatic fluorescence diagrams of Hela cells with repeated times of electroporation.

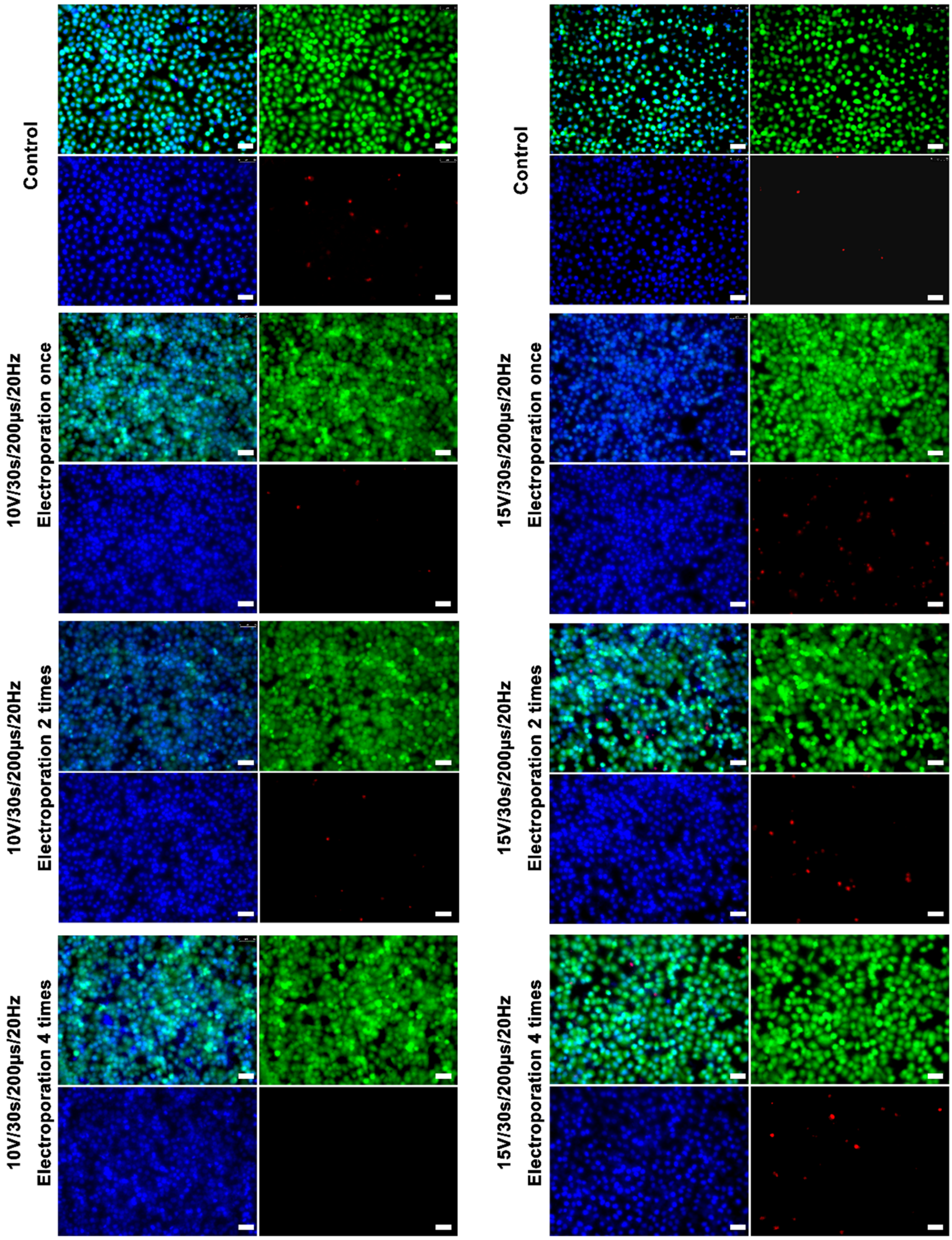

Figure S10. cells fluorescence diagrams after nanostraw-electroporation after 0-, 1-, 2- and 4-times nanostraw-electroporation under 10 V, 200 μs, 20 Hz, 30 s and 15 V, 200 μs, 20 Hz, 10 s. Live cells were stained by Calcein-AM (green fluorescence), dead cells were stained by PI (red fluorescence), cells' nucleus were stained with Hoechst 33342 (blue fluorescence). scale bar, 50 μm. Calcein-AM was excited at 496 nm wavelength, PI was excited at 535 nm wavelength, Hoechst 33342 was excited at excitation 364 nm wavelength.

S11, The detailed trichromatic fluorescence diagrams of Hela cells under four times of repeated electroporation under 10 V, 200 μs, 20 Hz, 30 s and control group without nanostraw-electroporation.

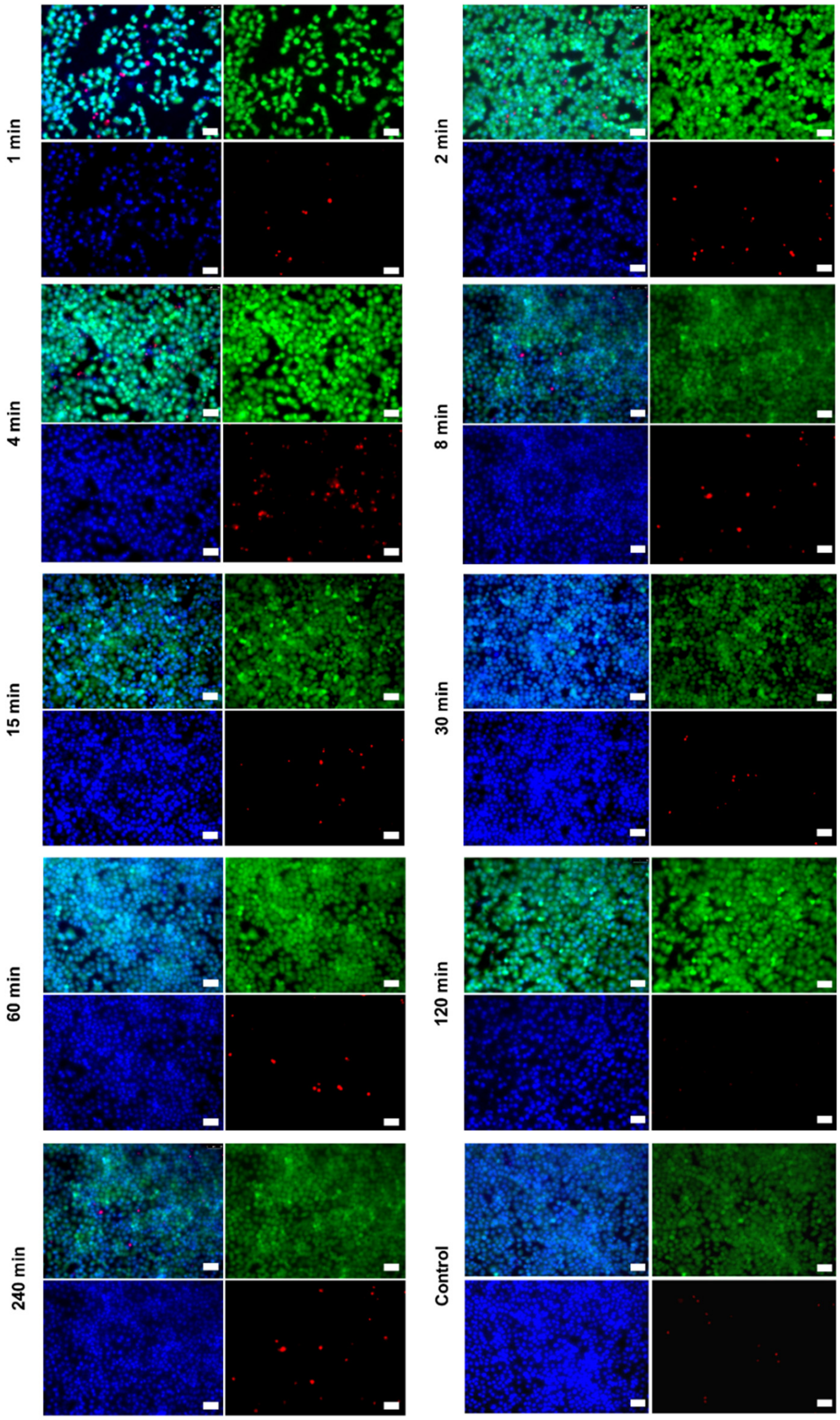

Figure S11. cells fluorescence diagrams after four times nanostraw-electroporation under 10 V, 200 μs, 20 Hz, 30 s with time intervals of, 1 min, 2 min, 4 min, 8 min, 15 min, 30 min, 60 min, 120 min, 240 min, and control group without nanostraw-electroporation. Live cells were stained by Calcein-AM (green fluorescence), dead cells were stained by PI (red fluorescence), cells' nucleus were stained with Hoechst 33342 (blue fluorescence). scale bar, 50 μm. Calcein-AM was excited at 496 nm wavelength, PI was excited at 535 nm wavelength, Hoechst 33342 was excited at excitation 364 nm wavelength.

S12, The detailed trichromatic fluorescence diagrams of Hela cells under four times of repeated electroporation under 15 V, 200 μs, 20 Hz, 10 s and control group without nanostraw-electroporation.

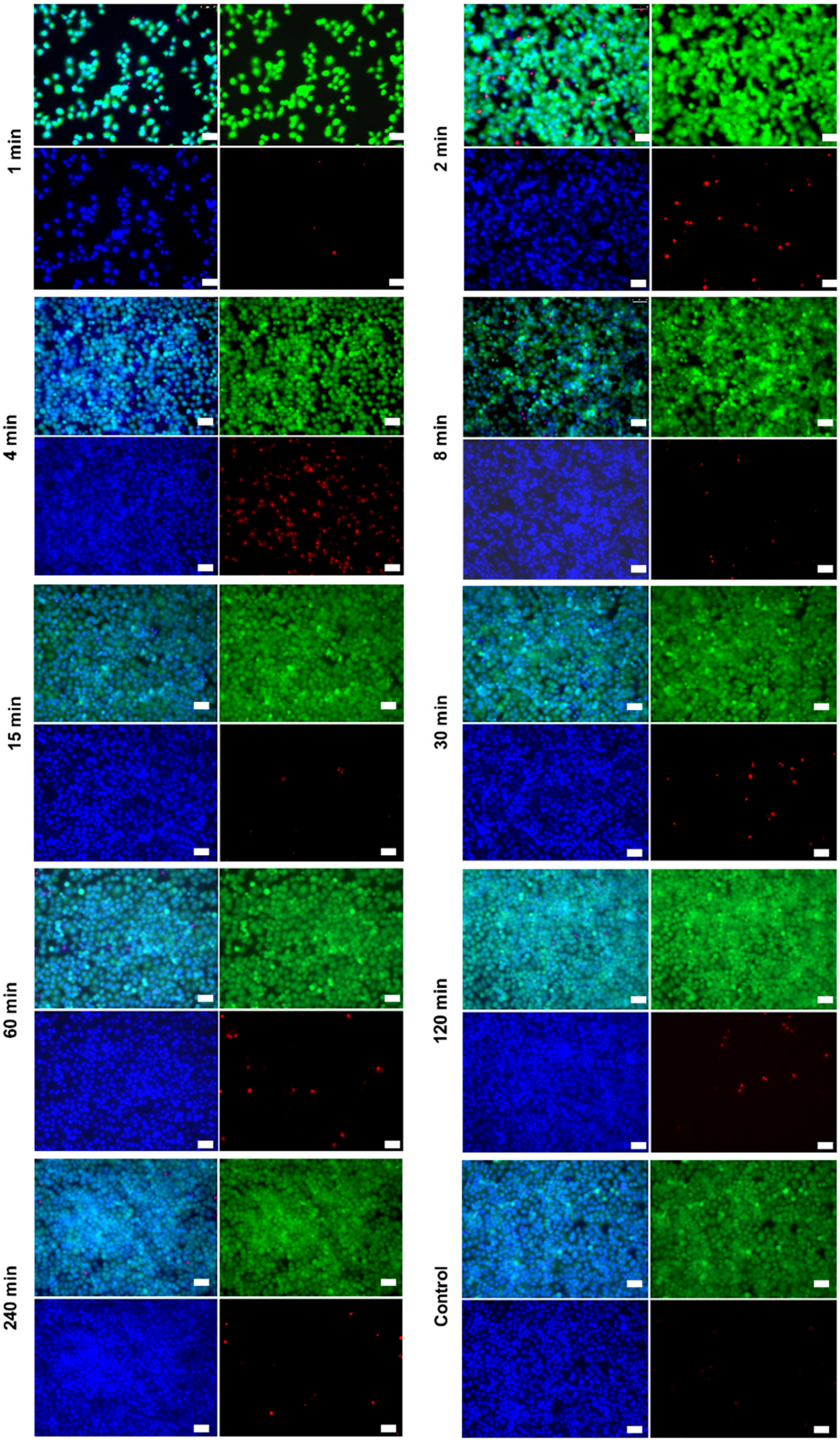

Figure S12. cells fluorescence diagrams after four times nanostraw-electroporation under 15 V, 200 μs, 20 Hz, 10 s with time intervals of, 1 min, 2 min, 4 min, 8 min, 15 min, 30 min, 60 min, 120 min, 240 min, and control group without nanostraw-electroporation. Live cells were stained by Calcein-AM (green fluorescence), dead cells were stained by PI (red fluorescence), cells' nucleus were stained with Hoechst 33342 (blue fluorescence). scale bar, 50 μm. Calcein-AM was excited at 496 nm wavelength, PI was excited at 535 nm wavelength, Hoechst 33342 was excited at excitation 364 nm wavelength.
